# Enterovirus-A71 exploits RAB11 to recruit chaperones for virus morphogenesis

**DOI:** 10.1186/s12929-024-01053-2

**Published:** 2024-06-28

**Authors:** Qing Yong Ng, Vikneswari Mahendran, Ze Qin Lim, Jasmine Hwee Yee Tan, Joel Jie Feng Wong, Justin Jang Hann Chu, Vincent T. K. Chow, Newman Siu Kwan Sze, Sylvie Alonso

**Affiliations:** 1 Infectious Diseases Translational Research Programme, Department of Microbiology and Immunology, Yong Loo Lin School of Medicine, National University of Singapore, Singapore, Singapore; 2 Immunology Programme, Life Sciences Institute, National University of Singapore, Singapore, Singapore; 3 Proteomics and Mass Spectrometry Services Core Facility, School of Biological Sciences, Nanyang Technological University, Singapore, Singapore; 4 Department of Health Sciences, Faculty of Applied Health Sciences, Brock University, St Catharines, ON L2S 3A1 Canada

**Keywords:** Enterovirus A71 (EV-A71), Hand, Foot and Mouth Disease (HFMD), RAB11, Replication organelle, CCT8, Chaperonin-containing T-complex (TRiC)

## Abstract

**Background:**

Enterovirus 71 (EV-A71) causes Hand, Foot and Mouth Disease (HFMD) in children and has been associated with neurological complications. The molecular mechanisms involved in EV-A71 pathogenesis have remained elusive.

**Methods:**

A siRNA screen in EV-A71 infected-motor neurons was performed targeting 112 genes involved in intracellular membrane trafficking, followed by validation of the top four hits using deconvoluted siRNA. Downstream approaches including viral entry by-pass, intracellular viral genome quantification by qPCR, Western blot analyses, and Luciferase reporter assays allowed determine the stage of the infection cycle the top candidate, RAB11A was involved in. Proximity ligation assay, co-immunoprecipitation and multiplex confocal imaging were employed to study interactions between viral components and RAB11A. Dominant negative and constitutively active RAB11A constructs were used to determine the importance of the protein’s GTPase activity during EV-A71 infection. Mass spectrometry and protein interaction analyses were employed for the identification of RAB11A’s host interacting partners during infection.

**Results:**

Small GTPase RAB11A was identified as a novel pro-viral host factor during EV-A71 infection. RAB11A and RAB11B isoforms were interchangeably exploited by strains from major EV-A71 genogroups and by Coxsackievirus A16, another major causative agent of HFMD. We showed that RAB11A was not involved in viral entry, IRES-mediated protein translation, viral genome replication, and virus exit. RAB11A co-localized with replication organelles where it interacted with structural and non-structural viral components. Over-expression of dominant negative (S25N; GDP-bound) and constitutively active (Q70L; GTP-bound) RAB11A mutants had no effect on EV-A71 infection outcome, ruling out RAB11A’s involvement in intracellular trafficking of viral or host components. Instead, decreased ratio of intracellular mature viral particles to viral RNA copies and increased VP0:VP2 ratio in siRAB11-treated cells supported a role in provirion maturation hallmarked by VP0 cleavage into VP2 and VP4. Finally, chaperones, not trafficking and transporter proteins, were found to be RAB11A’s top interacting partners during EV-A71 infection. Among which, CCT8 subunit from the chaperone complex TRiC/CCT was further validated and shown to interact with viral structural proteins specifically, representing yet another novel pro-viral host factor during EV-A71 infection.

**Conclusions:**

This study describes a novel, unconventional role for RAB11A during viral infection where it participates in the complex process of virus morphogenesis by recruiting essential chaperone proteins.

**Supplementary Information:**

The online version contains supplementary material available at 10.1186/s12929-024-01053-2.

## Introduction

After the near complete eradication of its close cousin Poliovirus, Enterovirus-A71 (EV-A71) has emerged as a public health concern, particularly among paediatric patients [1, 2]. EV-A71 belongs to the *Picornaviridae* family and, together with Coxsackievirus A16 (CVA16), is one of the main causative agents of Hand Foot Mouth Disease (HFMD). HFMD is highly infectious and typically affects children aged 5 and below, but also the elderly and immunocompromised individuals. Millions of HFMD cases are reported every year worldwide, with recurring outbreaks in the Asia Pacific region every 2 to 3 years [2]. Uncomplicated, self-limiting HFMD manifests as sore throat, fever, skin rash, ulcers in the mouth and blisters on the soles and palms. More rarely, HFMD may result in polio-like neurological symptoms, such as acute flaccid paralysis and encephalomyelitis that can be fatal or lead to long-term cognitive and motor deficits [3–5]. EV-A71 infections have been more frequently associated with such devastating complications granting this highly transmissible virus the status of one of the most medically concerning neurotropic virus.

EV-A71 is a non-enveloped virus that harbours a 7.4 kb single-stranded RNA genome within its capsid. The viral RNA encodes for a single polyprotein that is proteolytically cleaved into four viral structural proteins (VP1-VP4) and seven non-structural proteins (2A-2C, 3A-3D) [6]. EV-A71 strains are grouped under 11 sub-genotypes namely A, B1-B5, C1-C4, largely characterised by their highly variable VP1 sequences. New sub-genotypes, such as D and E, have been introduced over the years, illustrating the fast-evolving nature of EV-A71 genomes, fuelled by high mutation rate and recombination events among co-circulating strains [7, 8].

There is currently no effective therapeutic drug against HFMD [3]. Treatment is supportive in nature and often combined with public health measures such as school and playground closure to try and limit transmission [9]. Those measures force parents to take leave to look after their children, adding an economic dimension to this disease in affected societies. Three EV-A71 vaccines have been approved in China, and consist of formalin-inactivated C4 virus formulations, the predominant sub-genotype in China [10, 11]. The effectiveness of these monovalent vaccines however has yet to be evaluated outside China, in regions where the predominant circulating sub-genotype is not C4, and/or where several EV-A71 sub-genotypes co-circulate.

The lack of effective treatment options against HFMD in general and EV-A71 in particular, is mainly attributed to limited research efforts to understand the molecular mechanisms of pathogenesis. Specifically, identification of the viral determinants and host factors involved in EV-A71 fitness and virulence are key to the rational design of effective interventions. Using a proteomics approach, our group has previously reported two novel host factors, namely prohibitin-1 (PHB) and peripherin (PRPH) that are exploited by EV-A71 during its infection cycle in neuronal cells. While PHB was found to be closely associated with viral replication complexes located at the mitochondrial membrane [12], intermediate neurofilament PRPH was shown to support virus entry and viral genome replication through interactions with structural and non-structural viral components [13]. The latter study also led to the identification of small GTP-binding protein Rac1 as a druggable pro-viral host factor.

Pursuing our efforts to identify host factors involved in EV-A71 infection cycle, we have undertaken a siRNA screen that targeted 112 host genes encoding for proteins involved in intracellular membrane trafficking. Indeed, membrane trafficking pathways are commonly exploited by viruses for the transport of their viral components to various subcellular compartments for genome replication, protein translation, post-translational modifications, virus assembly and virus exit [14, 15]. EV-A71 has been known to induce massive membrane re-arrangements to support its replication, and several host proteins have been associated with those events [14]. Here, we have identified 21 hits that encode for pro-viral factors. Among which, knockdown of RAB11A expression resulted in the most significantly reduced viral titers, implying that this host factor plays an important role during EV-A71 infection cycle.

A member of the RAB family, RAB11A is a small GTPase involved in the exocytic and late endosomal recycling pathways. Similar to other RABs, RAB11 is activated when a guanine exchange factor (GEF) replaces the bound GDP with GTP. GTP-bound RAB11 interacts with its protein partners known as RAB11-family interacting proteins (FIPs), which subsequently allows RAB11 to engage with a series of other proteins that help RAB11-bound vesicles to move towards their destined subcellular location [16, 17]. Upon GTP hydrolysis, RAB11 then interacts with yet another group of proteins which promote activities such as membrane fusion [16]. Three isoforms of RAB11 have been reported, namely RAB11A, RAB11B and RAB25. RAB11A and RAB11B isoforms share 90% nucleotide sequence homology, while similarity with RAB25 is around 60%. All three isoforms are known to be involved in the recycling pathways, but they are also believed to have distinct cellular functions [18]. They are also differentially expressed whereby RAB11A is ubiquitously expressed, while RAB11B expression is limited to the brain, testis and heart, and RAB25 is restricted to the epithelial cells in lungs, colon and kidney [18].

Several viruses have been reported to exploit RAB11 during infection, with influenza virus being the most well studied [19–24]. RAB11 has been described to promote aggregation and transport at the plasma membrane of the eight viral RNA segments during influenza virus assembly step [19, 21]. In contrast, little is known about the involvement of RAB11 during EV-A71 infection. Current literature has suggested that RAB11 is exploited by enteroviruses for modulating the cholesterol level in replication organelles (RO) to create a microenvironment favourable for viral RNA synthesis [25]. Such cholesterol re-routing is associated to PI4KB, which is recruited to the ROs by viral protein 3A through GBF1 or ACBD3 proteins [25–29]. In this scenario, RAB11 is believed to facilitate the transport of extracellular/plasma membrane cholesterol towards the RO.

Here, we have combined various experimental approaches to dissect the role of RAB11 during EV-A71 infection, including genetics, biochemistry, dynamic confocal imaging and mass spectrometry techniques. Our data suggest that the main role of RAB11 during EV-A71 infection is to support the maturation and assembly of newly formed virus particles, rather than facilitating trafficking activities.

## Material and methods

### Cell lines and virus strains

Human rhabdomyosarcoma (RD) cell line (American Type Culture Collection (ATCC), CCL-136), mouse motor neuron-like hybrid cell line NSC-34 (CELLutions Biosystems, CLU140), and human neuroblastoma SH-SY5Y cell line (ATCC #CRL-2266) were maintained in Dulbecco’s Modified Eagle’s Medium (DMEM) supplemented with 10% fetal bovine serum (FBS) (GIBCO) at 37 °C with 5% CO2. Enterovirus-A71 (EV-A71) clinical isolates of sub-genotype B4 (strain S41, Genbank accession number AF316321), B5 (EVGP-18–254 strain,, Genbank accession number OQ571388), C1 (EVGP-18–331 strain, Genbank accession number OQ571387), C2 (C2 strain, Genbank accession number NUH0075/SIN/08), and CVA16 (CA16-G-10, Accession number U05876), were plaque purified and propagated in RD cells maintained in DMEM supplemented with 2% FBS. Harvested culture supernatants containing the virus particles were aliquoted and stored at -80 °C.

### siRNA knock-down (reverse transfection)

Appropriate dilutions of both siRNAs (Suppl. Table S1) and transfection reagents (Dharmafect-1 for knockdown in RD and NSC34 cells, and Dharmafect-4 for knockdown in SH-SY5Y cells) were performed in MEM-RS (Cytiva, Cat #SH30564.01). Equal volume of diluted siRNAs and transfection reagents were mixed and incubated for 30 min at room temperature (RT). After 30 min, 20 uL or 100uL of siRNA-Dharmafect mixture were added to each 24-well or 96-well respectively. 10^5^ cells in 400uL or 2.5 × 10^4^ cells in 80uL of culture medium were then added to each 24-well and 96-well respectively. The plate was incubated for 48 h at 37 °C, 5% CO2 before further analysis or treatments were performed.

### siRNA library screening

Mouse Membrane Trafficking siRNA library (Dharmacon, Cat # 115505) was reconstituted using DPEC-treated water (Invitrogen, Cat # AM9906). For each 96-well (Thermo Scientific, Cat #243656), 2.5 × 10^4^ NSC34 cells were reversed transfected with 50 nM of each siRNA pool from the library. At 48 h post transfection, the cells were infected with EV-A71 sub-genotype B4 Strain S41 at a multiplicity of infection (MOI) of 30, and the culture supernatants were collected at 48 h post-infection (h.p.i). The viral titers were determined by plaque assay and expressed as percentage of viral titer reduction compared to siRNA non-template control (NTC). Two independent screening campaigns were performed.

### Virus kinetics assay

Reverse siRNA-transfected cells were infected with S41 virus at MOI 0.1 (SH-SY5Y and RD cells), or MOI 30 (NSC34 cells). After two washes with 1X PBS, fresh 1X DMEM supplemented with 2% FBS was added to the cells, and the plates were further incubated at 37 °C, 5% CO_2_. At the indicated time points, the infected cells and their culture supernatants were harvested for Western blot analysis and plaque assay respectively. The culture supernatants were centrifuged at 4,000* g* for 10 min to remove any cells debris before the virus titers were determined by plaque assay. Concurrently, the cell monolayers were washed once with 1X PBS, and harvested by gentle scrapping. The three replicate wells were pooled together and centrifuged at 2,500* g* for 10 min. The cells pellets were resuspended in M-PER reagent (Thermo Scientific, Cat #78503) containing protease inhibitor (Thermo scientific, Cat #87Mouse Membrane Trafficking siRNA 786) and EDTA. After incubation on ice for 20 min, the lysates were centrifuged at 14,000* g* for 15 min, and the clarified lysates were stored at -20 °C for further analysis by Western blot.

### Cell viability assay

Cell viability was determined by AlamarBlue assay. Briefly, each well was washed once with 1X PBS prior to addition of AlamarBlue™ reagent (Invitrogen; Cat # DAL1025; diluted 1:10 in complete growth medium). The plates were then incubated at 37 °C, 5% CO_2_ for 1 h. Fluorescence signals were measured using Tecan SPARK plate reader (Ex570nm and Em585nm). The percentage of cell viability was determined in reference to untreated control.

### Western blot

Total protein content in cell lysates was determined by Bradford assay. 10ug of total protein per sample were mixed with reducing Laemmli buffer (Bio-Rad, Cat # 161074) and heated at 95 °C for 10 min. SDS gel electrophoresis was then run at 90 V for 2 h. For total protein content, precast stain-free gel (Bio-Rad, Cat #4568045) was imaged using Bio-Rad ChemiDoc. The proteins were next transferred onto nitrocellulose membrane (Bio-Rad, Cat #170427) using Bio-Rad Trans-Blot Turbo System. The membranes were then blocked in blocking buffer (5% blocking-grade milk in TBS-T) for 1 h, followed by overnight incubation with respective primary antibodies diluted in blocking buffer (Table S2). The membranes were washed thrice with TBS-T before incubation with the appropriate secondary antibodies diluted at 1:3,000 in blocking buffer for 1 h (Suppl. Table S2). The membranes were washed thrice in TBS-T before addition of chemiluminescent substrate (Thermo Scientific, Cat # 34076) and imaging using X-ray or Bio-Rad ChemiDoc. Images were analysed using ImageJ or ImageLab for semi-quantification.

### Plaque assay

RD cells were seeded in 24-well plates (10^5^ cells per well) and incubated overnight at 37 °C, 5% CO2. Clarified virus-containing culture supernatants were subjected to tenfold serial dilution in 2% FBS-DMEM, and 100uL of each dilution (10^–1^ to 10^–6^) were added per well. Three technical replicates were performed for each dilution. The cells were incubated for 1 h at 37 °C, 5% CO_2_. The cells were then washed twice with 1X PBS before overlaying with DMEM containing 1% carboxymethyl cellulose (Sigma Aldrich, Cat # 419303) and 2% FBS. The plates were incubated at 37 °C, 5% CO_2_ for 72 h before removing the overlay medium to fix and stain the cells using 0.02% crystal violet containing 4% PFA for 1 h. Plaques were scored visually at the appropriate dilution and viral titers were expressed as plaque forming units per mL (PFU/mL).

### Entry by-pass assay

EV-A71 S41 RNA was extracted from 400uL of infected culture supernatant using viral extraction kit (Qiagen, Cat # 52906). SH-SY5Y cells were transfected with siRAB11 mix, targeting both RAB11A and RAB11B isoforms. At 48 h post-transfection (h.p.t), 500 ng of purified viral RNA and 2uL of lipofectamine 2000 (Invitrogen, Cat #11668019) were each diluted in 50uL Opti-MEM (Gibco, Cat # 51985034), mixed together and incubated at RT for 30 min before being added to the siRNA-treated SH-SY5Y cells. The cells were incubated for another 24 h at 37 °C, 5% CO_2_ before the supernatants were harvested for viral titer determination by plaque assay.

### LucEV-A71 replicon and Bicistronic construct assays

*E. coli* strains harbouring LucEV-A71 replicon or bicistronic construct [30, 31] were cultured in LB broth containing 50ug/mL kanamycin (Gibco, Cat # 11815032). The plasmids were extracted using QIAprep Spin Miniprep Kit (QIAGEN, Cat # 27104) according to the manufacturer’s instructions.

LucEV-A71 assay: The plasmid was linearised using *Mlu*1 restriction enzyme (NEB, Cat # R3198S), and purified using Phenol:Chloroform:Isoamyl Alcohol (PCI 24:25:1; Sigma-Aldrich, Cat # 77617). In-vitro Transcription was performed using 1ug of linearised plasmid with MEGAscript® T7 Transcription Kit (Thermo Scientific, Cat # AM1334) according to the manufacturer’s protocol. The suspension was cleaned up using Phenol:Chloroform:Isoamyl (24:25:1 v/v/v) and chloroform (Sigma, Cat # C2432). SH-SY5Y cells, reverse-transfected with siRAB11 mix or siNTC, were incubated at 37 °C, 5% CO_2_ for 48 h. After 48 h, 500 ng of LucEV-A71 transcript and 2uL of lipofectamine 2000 (Invitrogen, Cat #11668019) were each diluted in 50uL Opti-MEM (Gibco, Cat # 51985034) and mixed together. The resulting 100uL of RNA-lipofectamine mixture was incubated at RT for 30 min before adding it to the siRNA-treated SH-SY5Y cells. The plate was incubated for another 24 h at 37 °C, 5% CO_2_ before measurement of luciferase activity using Nano-Glo® Luciferase Assay (Promega, Cat # N1110) and Tecan Spark plate reader.

Bicistronic assay: siRAB11-treated cells were incubated at 37 °C, 5% CO_2_ for 48 h. After 48 h, 500 ng of plasmid and 2uL of lipofectamine 2000 (Invitrogen, Cat #11668019) were each diluted in 50uL Opti-MEM (Gibco, Cat # 51985034) and mixed together. The resulting 100uL of DNA-lipofectamine mixture was incubated at RT for 30 min before adding it to the siRNA-treated SH-SY5Y cells. The plates were incubated for another 24 h at 37 °C, 5% CO_2_ before measurement of Renilla and Firefly luciferase activities using Dual-Glo® Luciferase Assay System (Promega, Cat #E2920) and Tecan SPARK plate reader. Results were expressed as FLuc:RLuc ratio.

### Real-time polymerase chain reaction (qPCR)

Total RNA was extracted using E.N.Z.A Total RNA Kit (Omega Bio-Tek, Cat # R6834-02) and its concentration was determined using nanodrop. 100 ng of total RNA was used to perform qPCR using iTaq Universal One-Step RT-qPCR kit (Biorad, Cat # 1725141). Briefly, 10uL of iTaq SYBR mastermix were mixed with 0.25uL of iScript reverse transcriptase and 0.6uL of 10uM of both forward and reverse primers for each reaction (Suppl. Table S3). Nuclease-free water was added to reach a final volume of 20uL, and the mixture was subjected to qPCR run using Applied Biosystems 7500 Real-Time PCR System, based on BioRad recommended parameters.

### Immunofluorescence assay (IFA) and proximity ligation assay (PLA)

SH-SY5Y cells (10^5^) were seeded onto coverslips placed into a 24-well plate and were incubated at 37 °C, 5% CO_2_ overnight. Where indicated, cells were first transfected with 500 ng of relevant plasmids (eGFP-RAB11A DN/CA/WT) using Lipofectamine™ 3000 (Invitrogen; Cat #L3000015) according to the manufacturer’s instructions. The cells were then incubated at 37 °C, 5% CO_2_ for 24 h, before infection with S41 virus at MOI 0.1. At 48 h.p.i, the cells were fixed with 4% PFA for 15 min at RT, permeabilized using 0.1% Tween-20/PBS for 15 min and washed thrice with 1X PBS.

IFA: The coverslips were blocked in 2% BSA in PBS for 1 h at 37 °C, followed by incubation for 1 h at 37 °C with the relevant primary antibodies (Table S4), which were diluted in blocking buffer. The coverslips were washed thrice with 1X PBS, followed by incubation with appropriate secondary antibodies (Table S4) for 1 h at 37 °C. For compartment staining, coverslips were incubated with the respective compartment markers for 1 h at 37 °C after washing thrice in 1X PBS. The coverslips were washed thrice again in 1X PBS, followed by incubation with Hoechst 33342 (Invitrogen, Cat # R37605) for 15 min at RT. The coverslips were then mounted onto microscope slides using DABCO (Sigma Aldrich, Cat # 10981-100ML) before being imaged either with Olympus IX81 fluorescence microscope or Olympus FV3000 Confocal microscope.

PLA: PLA was carried out using the Duolink™ In Situ Red Starter Kit Mouse/Rabbit (Sigma-Aldrich, Cat #DUO92101-1KT). Briefly, the coverslips containing fixed cells (as described above) were blocked using blocking buffer, followed by incubation with the appropriate mouse and rabbit antibodies (Table S3) at 37 °C for 1 h. The coverslips were then washed thrice with Buffer A, before incubation with the two PLA probes for 1 h at 37 °C. After three washes with Buffer A, Ligase was added and incubated for 30 min. The ligase was washed off with buffer A before incubation with polymerase for 100 min. The coverslips were then mounted onto microscope slides using Duolink™ In Situ Mounting Medium with DAPI, and the slides were imaged with Olympus IX81 or Olympus FV3000 Confocal microscope.

### Antibody labelling

For some PLA experiments, EV-A71 VP1 antibody (Abnova; MAB1255-M08) was labelled using mouse IgG1 Flexible Antibody Labelling Kit (Proteintech, Cat # KFA021). Briefly, 0.5 µg of antibody was mixed with 1µL of Flexlinker prior to topping up with Flexbuffer to a volume of 8µL. The mixture was incubated in the dark for 5 min at RT, quenched by adding 2µL of FlexQuencher, followed by another 5 min incubation in the dark at RT.

### Co-immunoprecipitation (Co-IP)

EV-A71 infected and uninfected SH-SY5Y cells were scrapped off T175 tissue culture flasks, and were centrifuged at 2,500* g* for 10 min. The cell pellets were washed once with 1X PBS before being lysed in MPER reagent containing protease inhibitors (Thermo Scientific; Cat # 87785) and 5 mM EDTA. The lysates were then spun down at 14,000* g* for 15 min. Dynabeads crossed-linked to the appropriate antibodies were added to the clarified lysates and incubated at 4 °C for 3 h. The beads were washed thrice in 1X PBS and subjected to elution using 1% SDS. The eluted fractions were then mixed with Laemmli buffer and heated at 95 °C for 10 min before being analysed by Western blot.

### Co-IP/mass spectrometry

Co-IP was performed as described above, with the elution performed by heating the beads in Laemmli buffer at 70 °C for 10 min. The eluted samples were then subjected to 8–20% gradient SDS-PAGE. The protein bands were then excised and subjected to in-gel trypsin digestion. The digested peptides were separated and analysed using Dionex Ultimate 3000 RSLCnano system coupled to a Q Exactive instrument (Thermo Fisher Scientific, MA, USA). Separation was performed on a Dionex EASY-Spray 75 μm × 10 cm column packed with PepMap C18 3 μm, 100 Å (Thermo Fisher Scientific) using solvent A (0.1% formic acid) and solvent B (0.1% formic acid in 100% ACN) at flow rate of 300 nL/min with a 60-min gradient. Peptides were then analyzed on a Q Exactive apparatus with an EASY nanospray source (Thermo Fisher Scientific) at an electrospray potential of 1.5 kV. Raw data files were processed and converted to mascot generic file (mgf) format using Proteome Discoverer 1.4 (Thermo Fisher Scientific). The mgf files were then used for protein sequence database search using Mascot algorithm to identify proteins. Further analysis of identified proteins were done by employing both PantherDB and StringDB platforms. The mass spectrometry proteomics data have been deposited to the ProteomeXchange Consortium via the PRIDE [32] partner repository with the dataset identifier PXD043870.

For each protein identified in the infected or uninfected samples, its emPAI value was first deducted from the emPAI value obtained with the corresponding IgG isotype control sample to account for background noise due to unspecific binding. The resulting value was further normalized by dividing it by the emPAI of RAB11A (which corresponds to the amount of RAB11A being pulled down during Co-IP). The normalised value for each protein in the infected sample was further divided by the normalised value for the same protein in the uninfected sample to obtain the fold change difference reflected in Suppl. Table S5.

### Over-expression of RAB11A DN/CA/WT in SH-SY5Y cells

eGFP-RAB11A dominant negative (DN) and constitutively active (CA) plasmids [21] were obtained from Madison University, USA. Plasmid harbouring eGFP-RAB11A-WT was obtained by mutagenesis of eGFP-RAB11A-DN construct with Phusion® High-Fidelity DNA Polymerase (Thermo Scientific, Cat # F530S) using primers as follows: Forward ggtgttggaaagaGtaatctcctgtct; Reverse agacaggagattaCtctttccaacacc, where the capital letter features the point mutation introduced). These plasmids were transformed into *E. coli*, which were cultured in LB broth supplemented with 50ug/mL of kanamycin. The plasmids were extracted using Qiagen HiSpeed Plasmid Maxi Kit. SH-SY5Y cells (1.5 × 10^7^) were seeded into a T175 flask and incubated at 37 °C, 5% CO_2_ overnight. Each T175 flask was transfected with mixtures consisting of 52.5ug of plasmid in 131.25uL of Lipofectamine 3000 and 87.5uL of p3000 reagent. The flasks were incubated at 37 °C, 5% CO_2_ for 24 h. At 24 h.p.t, cells were trypsinized and subsequently sorted using BD FACSAria Fusion cell sorter. The GFP^+^ sorted cells were seeded into a 48-well plate (7.5 × 10^4^ cells per well). Cells were allowed to rest at 37 °C, 5% CO_2_ for 24 h. The cells were then transfected with 50 nM siRAB11B and were further incubated at 37 °C, 5% CO_2_ for 48 h before infection with S41 at MOI 0.1. At 18 h.p.i, the supernatants and cells were harvested for viral titer determination by plaque assay and Western blot analysis, respectively.

### Statistical analyses

All statistical analyses were performed using GraphPad Prism 9.0. Mann–Whitney statistical test was performed for comparing independent data pairs, while Kruskal–Wallis H test was employed for the comparison between 3 or more independent groups. The degree of significance was indicated by asterisks with *p < 0.05, **p < 0.01, ***p < 0.001, ****p < 0.0001, ns: not significant.

## Results

### siRNA screening identifies RAB11A as a pro-viral factor during EV71 infection in motor neurons

**Fig. 1:**
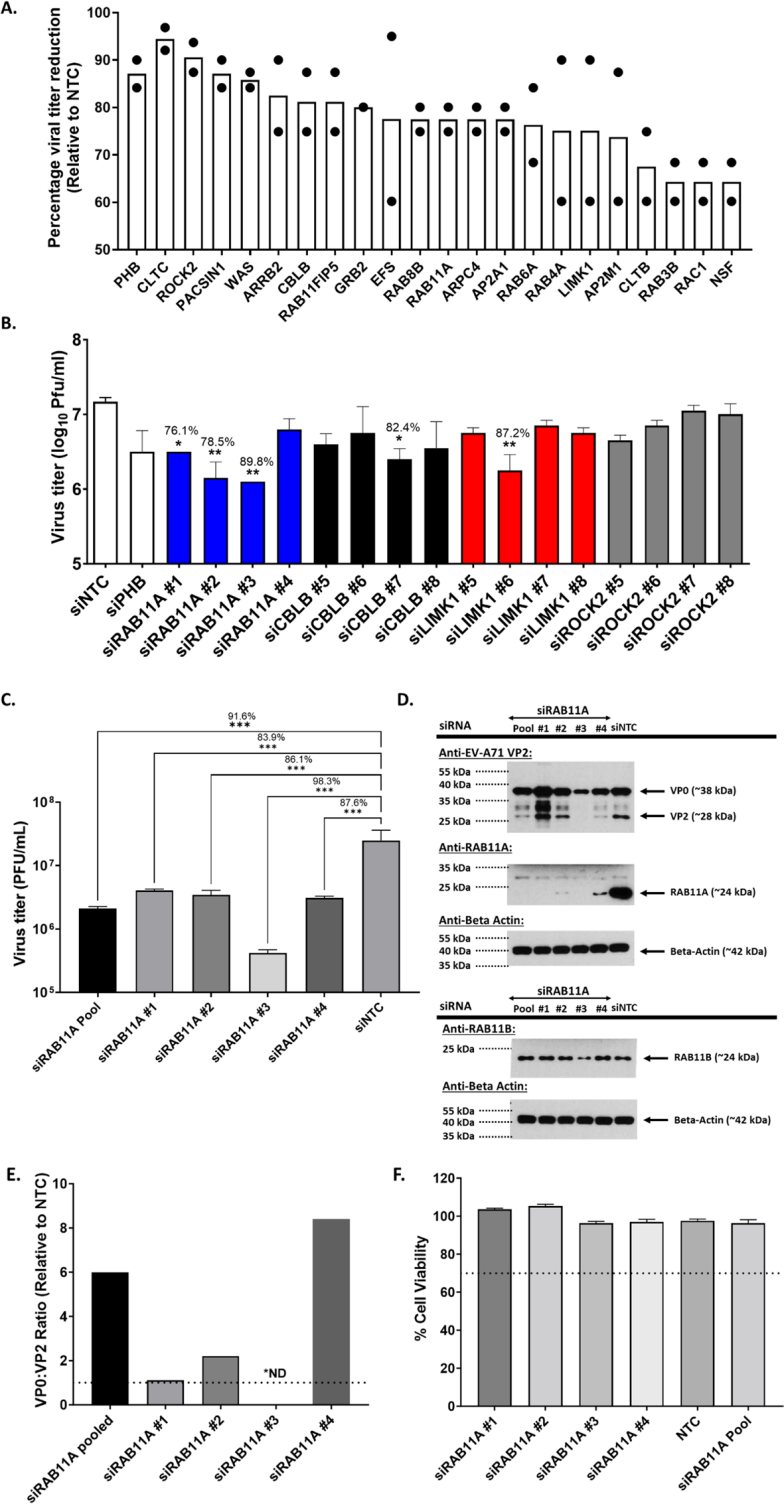
Identification of RAB11A as a pro-viral factor through screening of membrane trafficking siRNA library in NSC34 cells. **A** NSC34 cells were transfected with the siRNA library pools, then infected with EV-A71 S41 (MOI 30) 48 h later. At 48 h.p.i, the culture supernatants were harvested to determine the viral titers by plaque assay. Results are expressed as the percentage of virus titer reduction obtained for each siRNA treatment compared to Non-targeting siRNA non-targeting control (NTC). Genes with virus titer reduction > 50% in two independent screening experiments are shown. **B** NSC34 cells were transfected with deconvoluted siRNA targeting genes encoding RAB11A, CBLB, LIMK1 and ROCK2, followed by infection with EV-A71 S41 (MOI 30) 48 h later. At 48 h.p.i, virus titers in the culture supernatants were determined by plaque assay. **C**, **D** Further validation of RAB11A as a pro-viral factor using deconvoluted siRNAs. NSC34 cells were transfected with pool or deconvoluted siRNA targeting RAB11A, or with siNTC, followed by infection with EV-A71 S41 (MOI 30) at 48 h.p.t. The culture supernatants and cell lysates were harvested at 48 h.p.i. **C** Virus titers in the culture supernatants were determined by plaque assay. **D** The cell lysates were subjected to Western blot analysis using anti-VP2, anti-RAB11A, anti-RAB11B and anti B-actin antibodies. **E** VP0:VP2 ratio relative to NTC, based on signal band intensity measured by ImageJ. *ND, not determined. **F** AlamarBlue assay was performed on uninfected siRNA-treated cells at 48 h post-transfection. The readout for each treatment was relative to non-siRNA-treated cells to determine the percentage of cell viability. Percentage above 70% (indicated by the dotted line) was considered non-cytotoxic. Statistical analysis was performed for (**B**) and (**C**) using Kruskal–Wallis test against siNTC treatment (*p < 0.05, **p < 0.01, ***p < 0.001, ****p < 0.0001). The percentages of virus titer reduction for siRNA treatments that were statistically different from siNTC are indicated above the asterisk

Motor neuron-like NSC34 cells were subjected to a siRNA screen targeting 112 genes involved in membrane trafficking. We have previously described NSC34 cells as an in vitro infection model predictive of EV-A71 in vivo neurovirulence [33]. Viral titers in the culture supernatant were measured by plaque assay providing information on the overall effect of the siRNA-mediated knockdown (KD) on virus replication. Prohibitin-1 encoding gene (Phb) was used as positive control as we previously showed that PHB is involved in both entry and post-entry steps of EV-A71 infection cycle in NSC34 cells [12]. Non-targeting scramble siRNA control (NTC) was used as negative control. The screening was performed twice independently and a total of 21 hits were obtained that resulted in viral titer reduction greater than 50% compared to the siNTC-treated control (Fig. 1A). Among which, Rac1 was identified, which was previously reported by us as a pro-viral host factor during EV-A71 infection in neuronal cells [13], thereby validating our screen.

Four genes encoding for LIMK1, ROCK2, RAB11A and CBLB were selected for downstream validation using deconvoluted siRNA, based on their known physiological role in cytoskeleton (LIMK1 and ROCK2), recycling endosomal pathway (RAB11A) and proteasome degradation (CBLB). RAB11A KD resulted in significant viral titer reduction for three of the four siRNA species used (Fig. 1B). In contrast, KD of CBLB and LIMK1 expression led to viral titer reduction for only one siRNA species, while no significant viral titer reduction was seen with ROCK2 deconvoluted siRNAs. These data prompted us to select RAB11A for further characterization.

Deconvoluted siRAB11A KD experiment was repeated and further confirmed significant reduction in viral titers compared to siNTC, thus validating that RAB11A is a pro-viral host factor during EV-A71 infection in NSC34 cells (Fig. 1C). In addition, Western blot analysis of the cell lysates verified that RAB11A expression was effectively knocked down, which correlated with reduced VP2 signals except for siRNA#1, for which increased viral protein band intensity was observed and which may be due to some off-target effect (Fig. 1D). Interestingly, treatment with siRNA#3 was found to also reduce the expression of RAB11B isoform, which correlated with even greater reduction in viral titer (Fig. 1C) and VP2 signal (Fig. 1D), compared to the other siRNA-treated samples. This observation hence suggested that RAB11A and RAB11B isoforms may both be exploited by EV-A71 during its infection cycle in NSC34 cells. Interestingly, we also noticed that in RAB11 KD samples (except for siRNA#1), the ratio VP0:VP2 was clearly higher compared to siNTC control (Fig. 1E). Since virus maturation involves VP0 cleavage into VP2 and VP4, this observation hence suggested a potential role for RAB11A/B in this process.

### RAB11A and RAB11B isoforms are exploited by EV-A71 and CVA16 during infection in human cell lines

**Fig. 2:**
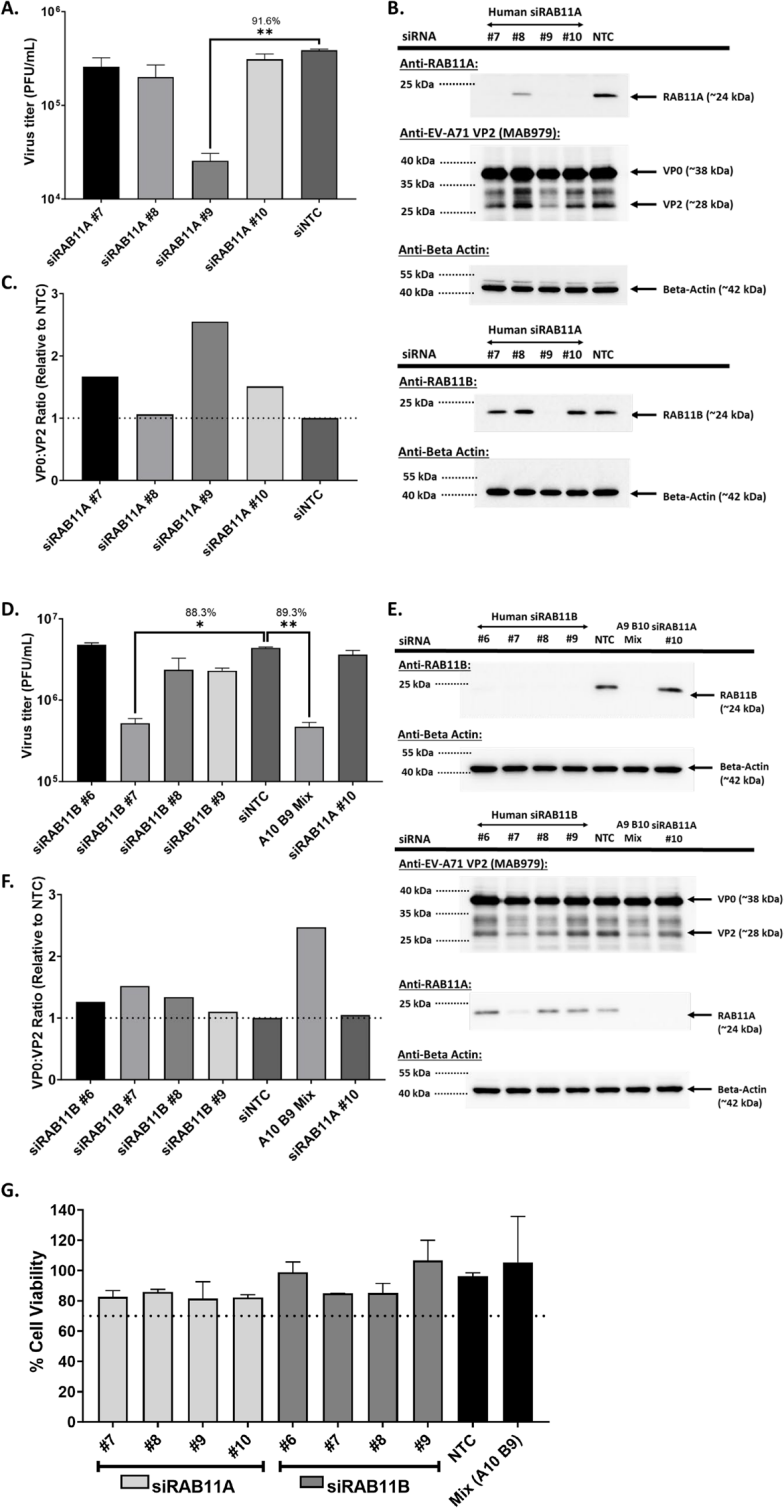
Effects of RAB11A and RAB11B KD on EV-A71 infection in RD cells. RD cells were transfected with deconvoluted human siRNA targeting RAB11A (**A**, **B**) or RAB11B (**C**, **D**). At 48 h.p.t, these cells were next infected with EV-A71 S41 (MOI 0.1). At 24 h.p.i, the culture supernatants and cell lysates were harvested. **A**, **D** Virus titers in the culture supernatants were determined by plaque assay. Statistical analysis was performed using Kruskal–Wallis test against siNTC treatment (*p < 0.05, **p < 0.01, ***p < 0.001, ****p < 0.0001). The percentages of virus titer reduction compared to NTC are indicated above the asterisk. **B**, **E** The cell lysates were subjected to Western blot analysis using anti-VP2, anti-RAB11A, anti-RAB11B and anti Bactin antibodies. Band intensities were normalised to Beta-actin and relative to NTC. **C**, **F** VP0:VP2 ratio, relative to NTC, based on signal band intensity measured by ImageLab were further determined. **G** AlamarBlue assay was performed on uninfected siRNA-treated cells at 48 h post-transfection. The readout for each treatment was relative to non siRNA-treated cells to determine the percentage of cell viability. Percentage above 70% (indicated by the dotted line) was considered non-cytotoxic

To further explore the role of RAB11A during EV-A71 infection, a similar siRNA KD approach was performed in human rhabdomyosarcoma (RD) cell line, which has been extensively employed to study EV-A71 pathogenesis. Unlike NSC34 cells, RAB11A KD in RD cells using RAB11A-specific siRNA pool and individual siRNA species did not lead to significant viral titer reduction (Fig. 2A), except for siRNA#9 for which significant reduction in both viral titers and VP2 signal were observed (Fig. 2A, B). Interestingly, treatment with siRNA#9 led to reduced expression in both RAB11A and RAB11B isoforms, whereas the other siRNA species impacted RAB11A expression only (Fig. 2B). This finding thus further supported the idea that both RAB11A and RAB11B may be exploited interchangeably by EV-A71 during infection. To confirm this hypothesis, RAB11B-specific KD was performed in RD cells. Consistently, only treatment with siRNA#7, which significantly reduced expression of both RAB11A and RAB11B, led to reduced viral titer in the culture supernatant, and reduced VP2 signal intensity in the cell lysates (Fig. 2D, E). To further demonstrate that co-KD expression of both RAB11A and RAB11B was responsible for reduced viral titer and VP2 intensity, RD cells were co-treated with siRAB11A #10 and siRAB11B #9 (A10B9 mix). While individual treatment with each siRNA species did not affect the viral titer and VP2 band intensity, co-treatment with both siRNA species led to significantly reduced viral titer and VP2 band intensity (Fig. 2D, E), hence demonstrating redundant functional role of RAB11A and RAB11B isoforms during EV-A71 infection. Furthermore, samples that were effectively knocked down for RAB11A and RAB11B, displayed higher VP0:VP2 ratio, hinting at a role for these proteins in VP0 cleavage (Fig. 2C, F). Similar observations were made in human neuroblastoma SH-SY5Y cells treated with siRAB11A#10, siRAB11B#9 or A10B9 mix (Suppl. Fig. S1).

We next investigated the importance of RAB11A/B during infection with EV-A71 strains representative of various EV-A71 sub-genogroups. SH-SY5Y cells were treated with siRAB11A#10, siRAB11B#9 or A10B9 mix before infection. Results indicated that significant reduction in viral titer and VP2 signal (and higher VP0:VP2 ratio) was observed in cells treated with A10B9 siRNA mix but not in cells treated with individual siRAB11A#10 or siRAB11B#9 (Suppl. Fig. S2). The same observations were made with Coxsackievirus A16 (CVA16), a close cousin of EV-A71 and another main causative agent of HFMD (Suppl. Fig. S2).

Taken together, the data supports that RAB11A and RAB11B isoforms were exploited by all major EV-A71 genogroups and CVA16 during infection, suggesting a conserved and important role for these proteins during infection. The consistent increase in VP0:VP2 ratio in RAB11-KD samples also suggested that RAB11 proteins may take part in EV-A71 maturation process.

### RAB11A/B is not involved in viral entry, viral genome replication, viral translation or virus exit, but influences virus maturation

To understand the role of RAB11A/B during EV-A71 infection cycle, various assays were performed. We first monitored over a 48-h period the viral titers in culture supernatant and VP0/VP2 protein expression in cell lysates treated with A10B9 mix (resulting in KD of both RAB11A and RAB11B) or siNTC. Significant viral titer reduction in the culture supernatant of siRAB11-treated samples was observed from 12 h.p.i onwards, and the magnitude of the reduction gradually increased over time till 48 h.p.i (Suppl. Fig. S3 panel A). Western blot analysis also clearly showed significant increase in the VP0:VP2 ratio in siRAB11-treated cell lysates compared to siNTC samples at all the time points post-infection (~ 80% increase) (Suppl. Fig. S3 panels B-D). We also probed for viral non-structural 3C and 3CD proteins in these cell lysates. The band signal intensity for VP0, 3C and 3CD proteins was comparable between siRAB11-treated and siNTC-treated cell lysates at the early time points post-infection that correspond to the first replication cycle (i.e. 6 and 12 h) (Suppl. Fig. S3 panels B, C). These observations thus suggested that RAB11A/B proteins do not affect the viral entry, viral protein translation, and viral genome replication, but specifically affect VP0 cleavage, hence virus maturation.

**Fig. 3:**
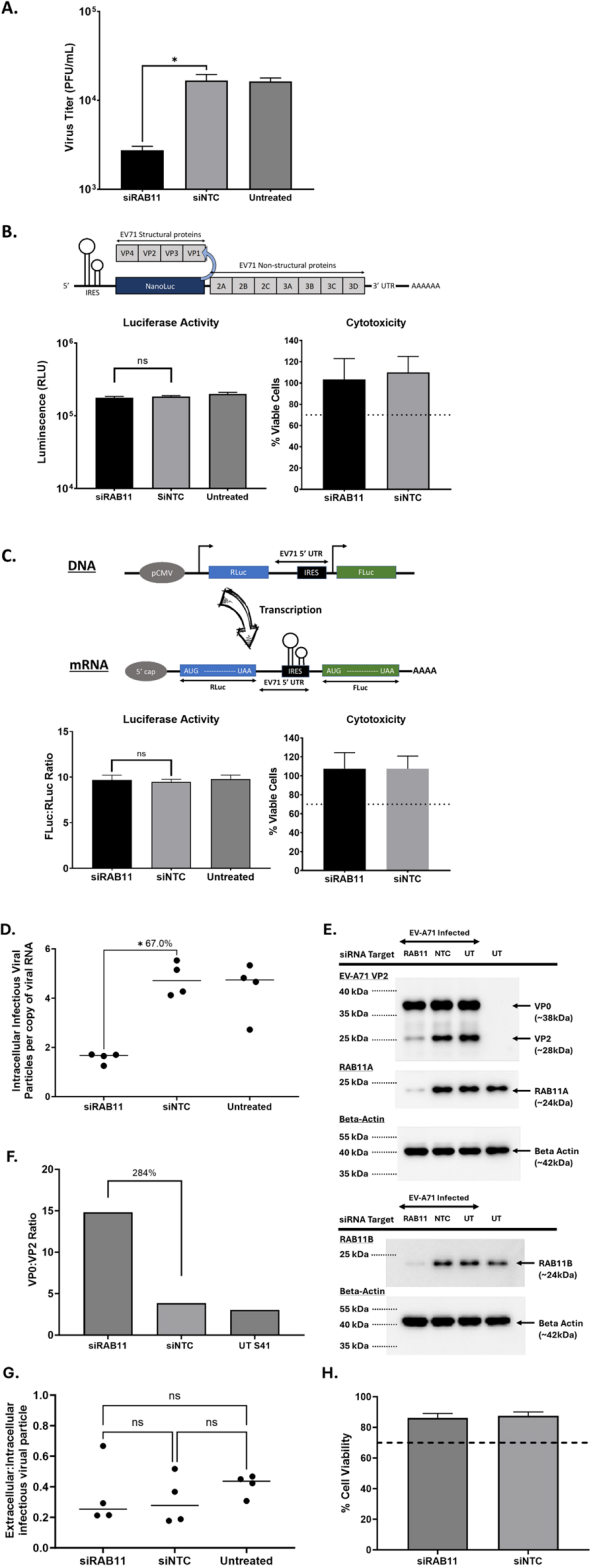
Role of RAB11 in viral entry, genome replication and viral protein translation. **A** Entry-bypass assay. SH-SY5Y cells were transfected with siRAB11 or siNTC, followed by transfection with 500 ng of EV-A71 S41 RNA genome. The culture supernatants were harvested 24 h post-transfection, and the viral titers were determined by plaque assay. **B** EV71-Luc replicon assay. SH-SY5Y cells were treated with siRAB11 or siNTC, then transfected with 500 ng of in vitro transcribed EV71-Luc RNA. The luciferase activity and cytotoxicity were measured at 24 h post-transfection. **C** EV71-Bicistronic reporter assay. SH-SY5Y cells were treated with siRAB11 or siNTC, then transfected with 500 ng of EV71 Bicistronic plasmid construct. Renilla and Firefly luciferase activities, and cytotoxicity (alamarBlue assay) were measured at 24 h post-transfection. **D-G** SH-SY5Y cells were transfected with siRAB11 or with siNTC, followed by infection with EV-A71 S41 (MOI 0.1) at 48 h.p.t. The culture supernatants and cells were harvested at 24 h.p.i. For each culture condition, the cells were split into 3 samples, centrifuged and the following experiments were performed: (**D**) One cell pellet was resuspended in PBS and subjected to two freeze–thaw cycles to release intracellular content. The resulting lysates were subjected to plaque assay to quantify the amount of intracellular infectious virus particles. The second cell pellet was subjected to total RNA extraction, and absolute quantification of the viral RNA copy number by qPCR. The ratio of intracellular viral titer to intracellular viral RNA copy number is shown. **E** The third cell pellet was lysed and subjected to Western blot analysis using anti-VP2, anti-RAB11A, anti-RAB11B and anti B-actin primary antibodies. **F** Quantification of VP0 and VP2 band intensities was performed using ImageLab. The VP0:VP2 ratio measured in siRAB11-treated, siNTC-treated and infected only samples is shown. **G** The amount of infectious viral particles in the culture supernatants was determined by plaque assay. The ratio of extracellular to intracellular infectious particles was calculated for each culture condition. **H** AlamarBlue assay was performed to assess cytotoxicity of siRNA treatment. The readout for each treatment was relative to untreated cells to determine the percentage of cell viability. Percentage above 70% (indicated by the dotted line) was considered non-cytotoxic. Statistical analysis was performed for (**A**-**D**, **G**) using Kruskal–Wallis test against siNTC treatment (*p < 0.05, **p < 0.01, ***p < 0.001, ****p < 0.0001). The difference in percentages between siRAB11 treatment and siNTC are indicated next to the asterisk

Next, we performed an entry bypass assay to further assess whether RAB11A/B was involved in the viral entry steps, which include receptor binding, internalization and virus uncoating. In this assay purified viral RNA genome was directly transfected into siRAB11-treated SH-SY5Y cells, thereby bypassing the entry steps. Significant viral titer reduction was still observed (Fig. 3A) with a magnitude of approx. 1 log, which is comparable to that observed upon natural infection (Suppl. Fig. S3A), hence suggesting that RAB11A/B had minimal role in the viral entry steps of EV-A71 infection cycle.

We next assessed the role of RAB11A/B in viral RNA genome replication specifically, using a Luc-EVA71 replicon where EV-A71 structural genes (P1 region) were replaced by NanoGlo luciferase encoding gene (Fig. 3B) [31]. siRAB11-treated SH-SY5Y cells were transfected with in vitro transcribed Luc-EVA71 RNA, and the luciferase signal was compared to siNTC-treated cells. No significant difference in signal intensity between siRAB11-treated and siNTC-treated cell cultures was observed (Fig. 3B), thus indicating that RAB11A/B is not involved in the initial round of viral RNA genome translation and replication by the viral RNA-dependent RNA polymerase 3D. This result was further supported by the absence of reduction in viral RNA levels measured at 4 h.p.i by qPCR in EV-A71-infected SH-SY5Y cells treated with siRAB11 (Suppl. Fig. S4A).

We also evaluated the impact of siRAB11KD on viral RNA genome translation by using a bi-cistronic construct, where a firefly luciferase-encoding gene is transcribed from a CMV promoter and contains EV-A71 cap-independent IRES for protein translation (Fig. 3C) [30]. No significant difference in luciferase activity was measured between siRAB11-treated and siNTC-treated cells (Fig. 3C), thus indicating that RAB11A/B did not contribute to IRES-dependent translation. To further assess the role of RAB11A/B proteins on viral protein translation, siRAB11-treated cells transfected with Luc-EVA71 replicon were treated with guanidine hydrochloride (GuHCl), an inhibitor of EV-A71 RNA synthesis [34–36]. In this setup, the luminescence signal measured in drug-treated cells would mostly arise from the IRES-dependent translation of the transfected RNA replicon construct. A significant reduction in relative luminescence units (RLU) was measured in drug-treated samples compared to vehicle-treated samples (Suppl. Fig. S5A), thus confirming the inhibitory effect of the drug on RdRp-mediated RNA replication. However, there was no significant difference between siRAB11-treated and siNTC-treated samples that were both treated with the drug (Suppl. Fig. S5A); and consistently, the RLU ratio between drug-treated and vehicle-treated were also comparable between siRAB11-treated and siNTC-treated cells (Suppl. Fig. S5B). These data thus further supported that RAB11 was not involved in EV-A71 IRES-mediated translation.

Together, the data indicated that RAB11A/B is neither involved in the viral entry step, nor does it contribute to viral genome replication and translation activities.

To investigate the role of RAB11A/B in virus maturation, we next quantified by plaque assay the amount of infectious (hence mature) viral particles that were present inside intact infected cells normalized to the amount of intracellular viral RNA copy number (quantified by qPCR). We reasoned that should RAB11A/B proteins be involved in the virus maturation process, we should observe lower infectious viral particles to viral RNA ratio in siRAB11-treated cells compared to siNTC control and untreated infected cells. The data showed that there was indeed significantly lower number of infectious viral particles per viral RNA copy in siRAB11-treated cells compared to siNTC-treated cells (Fig. 3D), hence supporting that RAB11 proteins play a role in the virus maturation process. We also confirmed that in siRAB11-treated infected cells, the VP0:VP2 ratio was significantly increased compared to the other groups (Fig. 3E, F). Lastly, we quantified by plaque assay the amount of infectious viral particles in the supernatant of these cultures and determined the ratio between infectious viral particles inside the cells and infectious viral particles released in the culture supernatants. The results indicated that there was no significant difference between siRAB11-treated cells, siNTC and untreated cells (Fig. 3G), thus supporting that RAB11 protein is not involved in the exit process of infectious viral particles.

Therefore together, results from these experiments allowed us to propose that RAB11A/B proteins are involved selectively in the viral maturation process that takes place intracellularly.

### RAB11A/B interacts with viral structural and non-structural proteins at the replication organelles

**Fig. 4:**
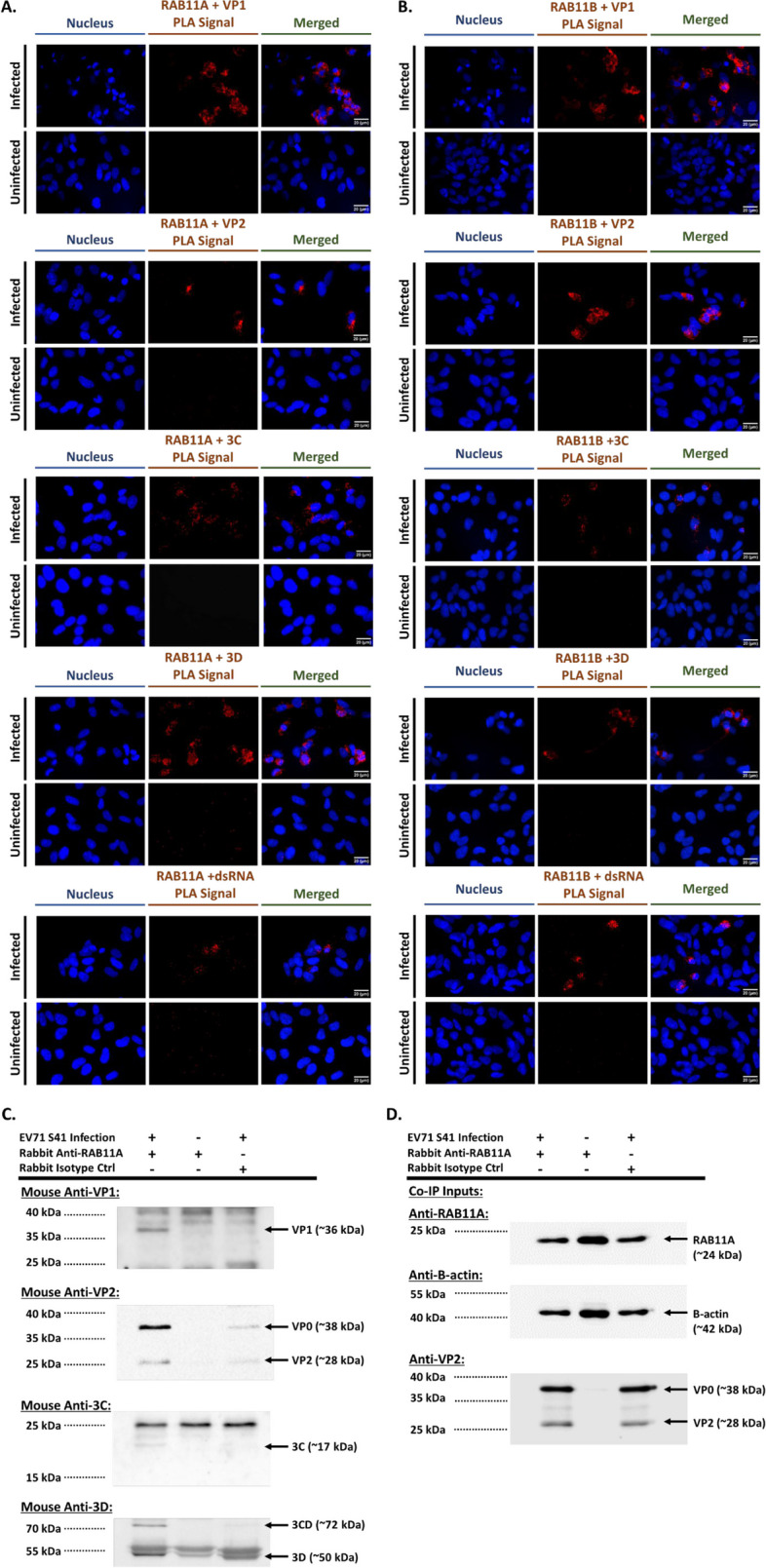
Co-localization and interaction of RAB11A and RAB11B with viral components. **A**, **B** Proximity ligation assay (PLA). SH-SY5Y cells were infected with EV-A71 S41 (MOI 0.1). At 24 h.p.i., the cells were fixed and permeabilized before staining with anti-RAB11A (**A**) or with anti-RAB11B (**B**), and anti-VP2, anti-VP1, anti-3D, anti-3C or anti-dsRNA antibodies. The cells were further stained with secondary antibodies conjugated with DNA probes. Ligation and polymerase chain reaction were then carried out for signal amplification. Nuclei were stained with DAPI. Images were captured at 60X magnification under Olympus IX81 microscope. **C**, **D** Coimmunoprecipitation (Co-IP). SH-SY5Y cells were infected with EV-A71 S41 (MOI 0.1). At 24 h.p.i., the cells were lysed for immunoprecipitation using anti-RAB11A or rabbit IgG isotype control monoclonal antibodies. **C** The pulldown samples were subjected to Western blot analysis using anti-VP1, anti-VP2, anti-3C and anti-3D antibodies. **D** Infected and uninfected cell lysates (Co-IP inputs) were analysed by Western blot prior to Co-IP

To gain further insights into the role of RAB11A/B during EV-A71 infection cycle, we investigated physical interactions between RAB11A/B and viral components by performing Proximity Ligation Assay (PLA). Results showed that both RAB11 isoforms co-localised with all the viral components tested including VP1, VP2, 3C, 3D and dsRNA (Fig. 4A, B). Furthermore, pull-down experiments using anti-RAB11A antibody showed that VP0, VP1, VP2, 3C, 3D, and 3CD were detected in the RAB11A pulldown samples (Fig. 4C), thus confirming close interactions between RAB11A protein and both structural and non-structural viral proteins.

**Fig. 5:**
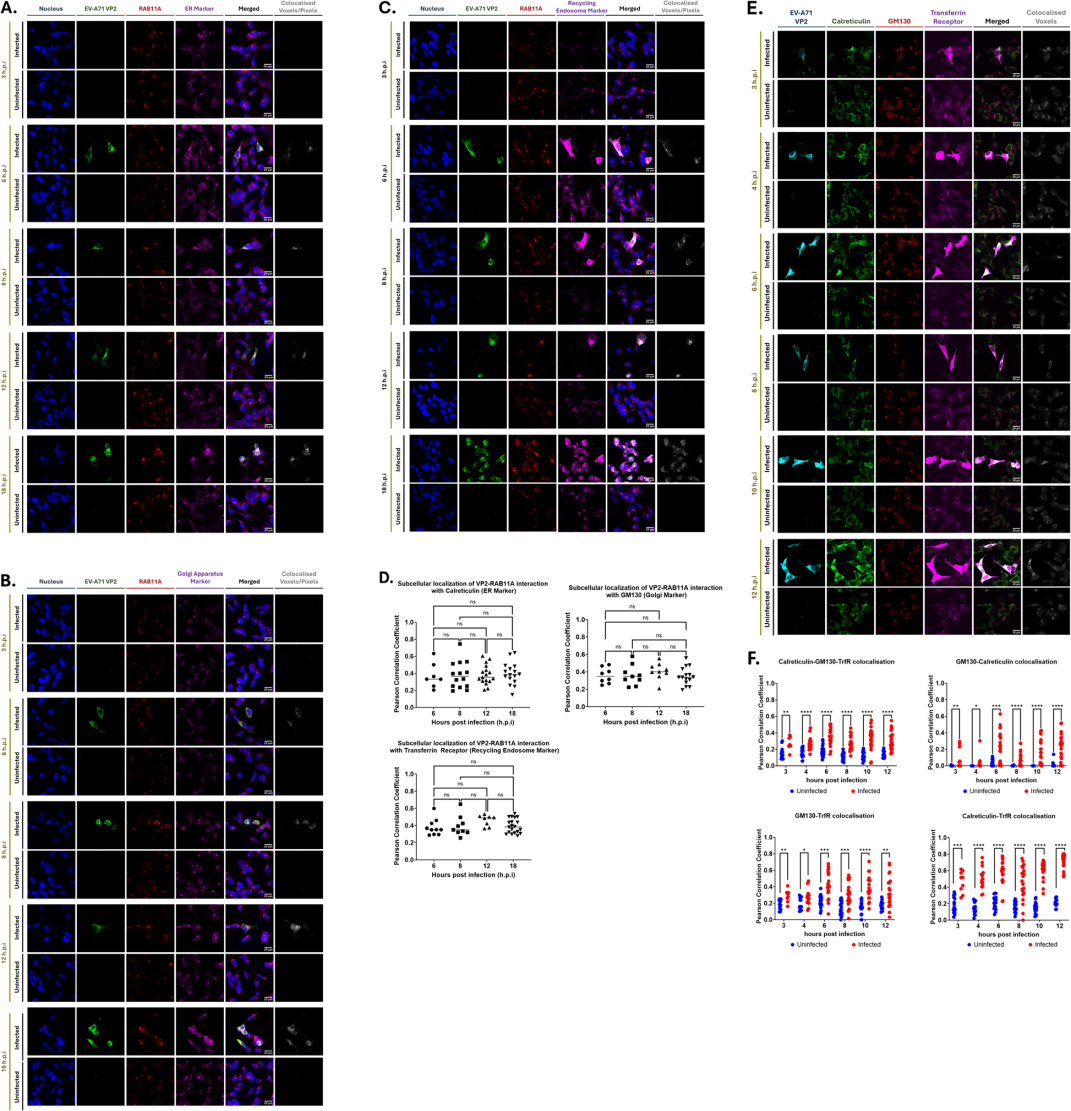
Colocalization between RAB11A and VP0/VP2 in various subcellular compartments, and colocalization of various compartment markers during EV-A71 infection. SH-SY5Y cells were infected with EV-A71 S41 (MOI 0.1). At the indicated time points post-infection, the cells were fixed and permeabilized, followed by staining with anti-RAB11A and anti-VP2 primary antibodies, and labelled secondary antibodies. Cells were then further stained with antibodies specific to compartment markers calreticulin for endoplasmic reticulum (**A**), GM130 for Golgi apparatus (**B**) and transferrin receptor (Tfr) for small recycling endosomes (**C**) prior to DAPI staining. Confocal images were captured under 100X objective and were analyzed using Fiji software to determine the voxels/pixels that represent three channels (green, red, magenta) co-localization. Briefly, a mask representing the co-localization of RAB11A and each viral component was delineated and subsequently overlayed with compartment marker signals to generate the ‘co-localized voxels’ images shown on the far right column. **D** Pearson correlation coefficient (PCC) values were computed using Fiji software. PCC value of 0 = no co-localization; 0.1 – 0.3 = weak co-localization; 0.3 – 0.5 = moderate co-localization; 0.5–1 = strong co-localization. Statistical analysis was performed using Kruskal–Wallis test with Dunn correction against siNTC treatment (*p < 0.05, **p < 0.01, ***p < 0.001, ****p < 0.0001). **E**, **F** SH-SY5Y cells were infected with EV-A71 S41 at MOI 0.1. **E** At the indicated time points post-infection, the cells were fixed and permeabilized, followed by staining with anti-VP2 primary antibody. Cells were then further stained using anti-calreticulin, anti-GM130, and anti-transferrin receptor antibodies. Images were captured using Olympus FV3000 at 100X objective lens. **F** Pearson Coefficient Correlation Analysis. Pearson correlation coefficient values were computed using Fiji software. PCC values between all three compartment markers or in a pairwise manner as indicated, were computed. PCC value of 0 = no colocalization; 0.1 – 0.3 = weak colocalization; 0.3 – 0.5 = moderate colocalization; 0.5–1 = strong colocalization. Statistical analysis was performed using Kruskal–Wallis test with Dunn correction against siNTC treatment (*p < 0.05, **p < 0.01, ***p < 0.001, ****p < 0.0001)

Since RAB11A/B are known to be involved in the exocytic and late endosomal recycling pathways, we hypothesized that these proteins may help transport the various viral components to their destined subcellular locations, for viral particle assembly and maturation. To address this hypothesis, multiplex confocal imaging was performed at various time points post-infection to track the dynamic interactions between RAB11A and viral proteins (VP2/VP0 and 3C/3CD) in various subcellular compartments. Results showed that interaction between RAB11A and VP2/VP0 could be detected from 6 h.p.i. onwards in the endoplasmic reticulum (ER), Golgi apparatus (GA), and small recycling endosomes (Fig. 5). Furthermore, Pearson Correlation Coefficient (PCC) values were determined to quantify the strength of co-localization between RAB11A and the viral proteins of interest, whereby the higher the PCC values the stronger the co-localization between targets. Results indicated that co-localization between RAB11A and VP2/VP0 was moderate and constant across the various subcellular compartments and across time (Fig. 5D). Similar observations were made when looking at the interactions between RAB11A and 3C/3CD (Suppl. Fig. S6), as well as the interactions between RAB11A and VP1, VP2, 3D, 3C or dsRNA (Suppl. Fig. S7).

Altogether, this multiplex confocal imaging approach supported that during EV-A71 infection RAB11A interacted with all the viral components tested, as early as 6 h.p.i and across three major subcellular compartments. However, extensive remodelling of the ER and GA membranes during EV-A71 infection has been reported to form the replication organelles (RO), where major events of viral replication as well as viral morphogenesis occur [37]. It is hence likely that the observed RAB11A interactions with viral components may occur at the RO, and not at the ER and GA per se. To test this hypothesis, we analysed the co-localization between the three compartment markers during the course of infection. Cells were stained with a VP2 antibody to differentiate infected from uninfected cells in the culture. Confocal fluorescence images and PCC analysis indicated that at early time points during infection (before 6 h.p.i.) and in uninfected cells, minimal co-localization was observed between calreticulin, GM130 and Transferrin Receptor (Fig. 5E, F). In contrast, from 6.h.p.i onwards, once VP0/VP2 can be readily detected, clear co-localization of the three markers was detected in the infected cells (Fig. 5E, F). This finding therefore supported extensive intracellular membrane remodelling during EV-A71 infection, where ER and GA merge to facilitate RO formation. Consequently, we concluded that interactions between RAB11A and the various viral components were likely to take place at the RO rather than in respective subcellular compartments.

### Role of RAB11 during EV-A71 infection is independent of its GTPase activity

Like all members of the RAB family, RAB11A/B contains a small GTPase domain that acts like a switch to cycle RAB11 between active (GTP-bound) and inactive (GDP-bound) states. Depending on its state, RAB11 interacts with distinct downstream effectors, driving distinct biological functions. Dominant negative (S25N; GDP-bound) and constitutively active (Q70L; GTP-bound) RAB11A mutants have been previously employed to study movement and fusion of RAB11A-containing membrane/vesicles [21, 38, 39]. Hence, we used these RAB11A mutants to assess the importance of RAB11A GTPase activity during EV-A71 infection.

SH-SY5Y were transfected with eGFP-RAB11AWT, eGFP-RAB11AS25N and eGFP-RAB11AQ70L plasmid constructs and were FACS sorted, thereby enriching the cultures in transfected cells between 79 to 89%. GFP + cells were then treated with siRAB11B#9 (to specifically knockdown RAB11B expression) and infected with EV-A71.

Confocal imaging of eGFP signal in uninfected cells confirmed the cellular distribution patterns of RAB11A WT and mutants as previously described [21, 39–42]. Briefly, as RAB11AS25N is GDP-bound, majority is associated with RAB-GDI and remains soluble within the cytoplasm [41, 43], thereby resulting in a diffused GFP signal across the cytoplasm (Suppl. Fig. S8). In addition, it was reported that RAB11AS25N localised at the trans-Golgi network (TGN) forming a dense perinuclear staining pattern [24, 41, 44], and this was also observed in our experiment (Suppl. Fig. S8, white arrow). In contrast, GTP-bound RAB11AQ70L was expected to localise to vesicle membranes [21, 42, 45], resulting in a punctate pattern (Suppl. Fig S8, white arrows). Moreover, RAB11AWT and RAB11AQ70L displayed comparable subcellular localisation pattern (Suppl. Fig. S8), as previously observed [40, 42, 45].

In EV-A71-infected cells, however, redistribution of all three overexpressed eGFP-RAB11 proteins (WT, S25N and Q70L) was observed across the cytoplasm, resulting in the loss of unique intracellular distribution patterns of these mutants as seen in uninfected cells (Suppl. Fig. S8).

**Fig. 6:**
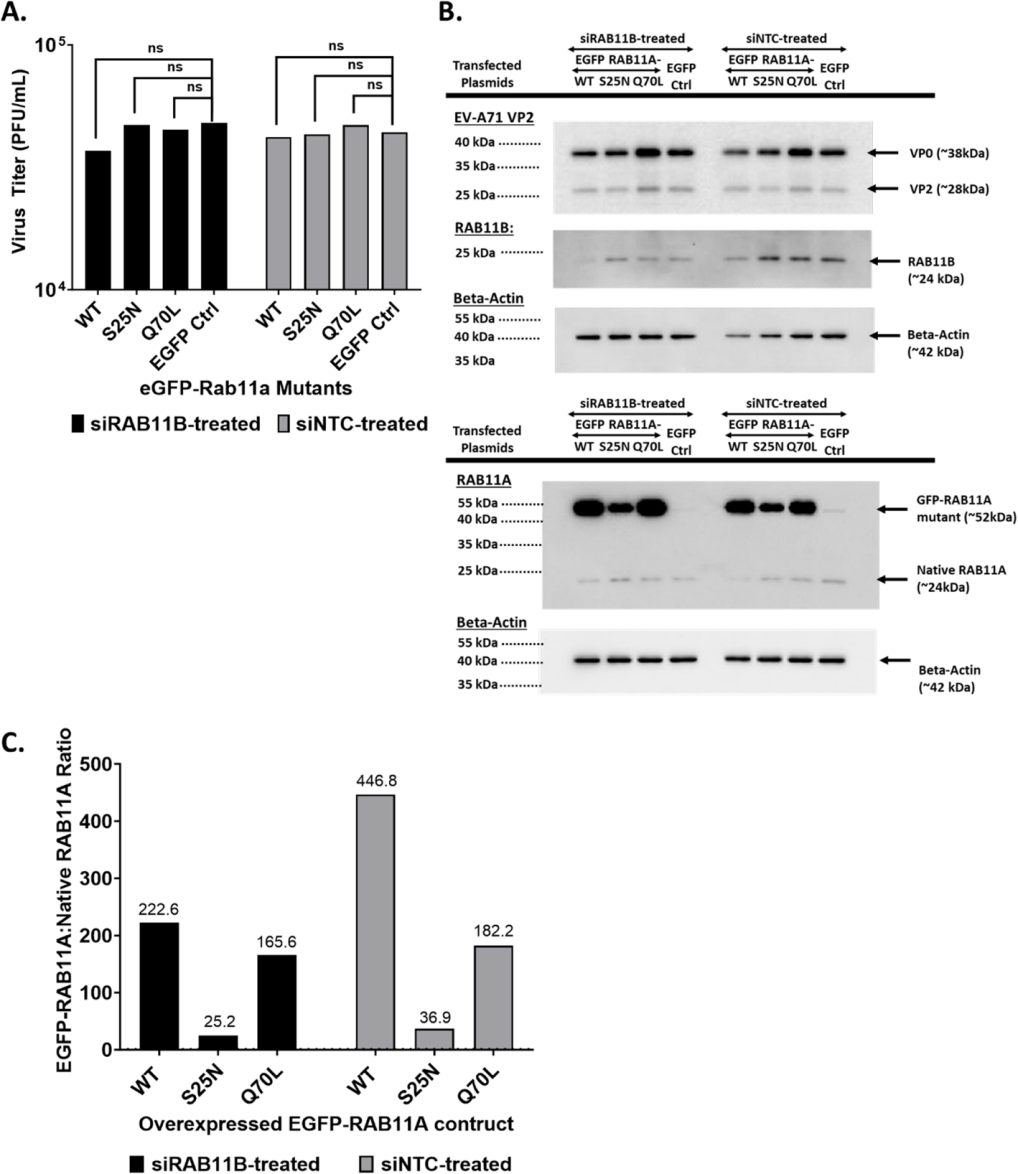
Effect of RAB11A DN and RAB11A CA overexpression on EV-A71 infection in siRAB11B KD SH-SY5Y cells. SH-SY5Y cells transfected with plasmids encoding for RAB11AWT, RAB11AS25N (dominant negative, DN), RAB11AQ70L (constitutively active, CA) and EGFP control, were FACS sorted (GFP +). The GFP + enriched cell suspensions were then transfected with siRAB11B #9 before infection with EV-A71 S41 (MOI 0.1). At 24 h.p.i, the culture supernatants and cell lysates were harvested. **A** Viral titers in culture supernatants were determined by plaque assay. Statistical analysis was performed using Mann–Whitney test against EGFP control (*p < 0.05, **p < 0.01, ***p < 0.001, ****p < 0.0001). **B** The cell lysates were analysed by Western blot using antiRAB11A or anti-VP2 primary antibody. Beta Actin was probed for normalization purpose. **C** Ratio of eGFPRAB11A to native RAB11A, based on band intensity measured by ImageLab

When looking at the viral titers and VP2 signal intensity, we observed that there was no significant difference between cells overexpressing RAB11AWT, RAB11AQ70L, and RAB11AS25N and that they were all comparable with siNTC-treated cells (Fig. 6). These observations hence supported that RAB11A GTPase activity was dispensable during EV-A71 infection.

Lastly, confocal imaging and PCC analysis indicated comparable co-localization patterns and strengths between RAB11AWT, RAB11AS25N and RAB11AQ70L, with viral components (VP1, VP2, 3C, 3D, and dsRNA) (Suppl. Fig. S9).

Together, these observations failed to support a differential outcome when over-expressing RAB11AWT, RAB11AS25N or RAB11AQ70L during EV-A71 infection, thus supporting that EV-A71 exploited RAB11A regardless of its GTPase activity status. This further implies that EV-A71 did not exploit the trafficking function of RAB11 to shuttle viral proteins to various subcellular locations, a function that requires RAB11 GTPase activity [16].

### EV-A71 re-directs RAB11A interactions with chaperones

**Fig. 7:**
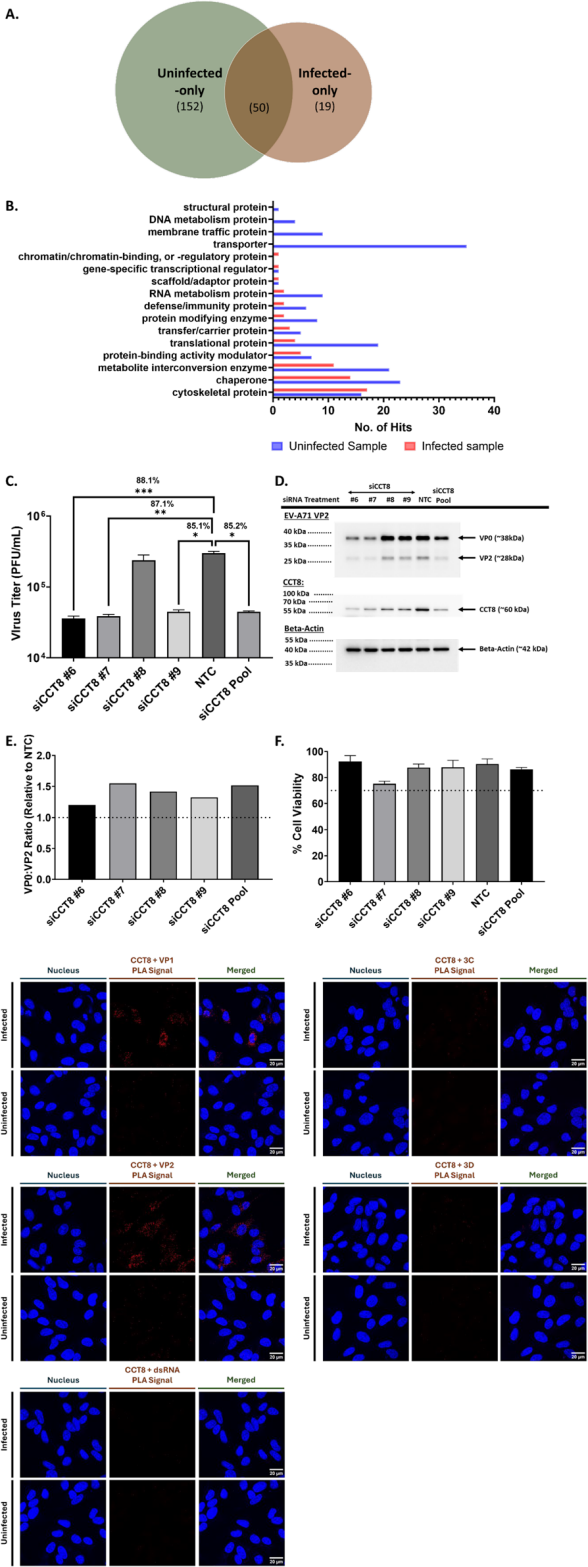
RAB11A interacting partners during EV-A71 infection. SH-SY5Y cells were infected with EV-A71 S41 (MOI 0.1). At 24 h.p.i, cell lysates were pulled down with antiRAB11A antibody. The proteins were identified by mass spectrometry. **A** Venn diagram. **B** PantherDB classification of proteins only found or enriched in pulldowns from infected or uninfected samples. **C**, **D** SHSY5Y cells were transfected with deconvoluted siRNA targeting CCT8 or siNTC. At 48 h.p.t, cells were subjected to infection with EV-A71 S41 (MOI 0.1). At 24 h.p.i, the culture supernatants and cells were harvested. Virus titers in the cultured supernatants were determined by plaque assay (**C**). The percentage of viral titer reduction compared to NTC was indicated above the asterisk. Statistical analysis was performed using Kruskal–Wallis test against siNTC treatment (*p < 0.05, **p < 0.01, ***p < 0.001, ****p < 0.0001). Cell lysates were subjected to Western blot analysis using anti-VP2, anti-RAB11A, anti-CCT8 and anti B-actin antibodies (**D**). **E** VP0:VP2 ratio relative to NTC, based on band intensity measured by ImageLab. **F** AlamarBlue assay was performed in uninfected cells to assess cytotoxicity of siRNA treatments at 48 h post-transfection. The readout for each treatment was relative to non siRNA-treated cells to determine the percentage of cell viability. Percentage above 70% (indicated by the dotted line) was considered non-cytotoxic. **G** Co-localization and interactions of CCT8 with viral components were assessed by proximity ligation assay (PLA). SH-SY5Y cells were infected with EV-A71 S41 (MOI 0.1). At 24 h.p.i., the cells were fixed and permeabilized before staining with anti-CCT8, paired with anti-VP2, anti-VP1, anti-3D, anti-3C or anti-dsRNA antibodies. The cells were further stained with secondary antibodies conjugated with DNA probes. Ligation and polymerase chain reaction were then carried out for signal amplification. Nuclei were stained with DAPI. Images were captured at 100X magnification under Olympus FV3000 confocal microscope

To further characterize the role of RAB11A/B during EV-A71 infection, mass spectrometry of RAB11A pulldown from infected and uninfected samples was performed to identify RAB11A interacting partners (Suppl. Fig. S10). A total of 69 proteins were identified from the infected pulldown samples, far lesser than the 221 hits identified from the uninfected samples, with infected and uninfected samples sharing 50 common candidates (Fig. 7A). Importantly, 19 of the 69 proteins found in infected samples were unique and not found in the uninfected pulldowns. These observations thus indicated that interactions between RAB11A and its host partners were significantly altered during EV-A71 infection. PantherDB classification revealed that the number of transporter proteins and proteins involved in membrane trafficking was greatly reduced compared to uninfected sample (Fig. 7B), further validating that RAB11 was not exploited for its trafficking function during EV-A71 infection. Interestingly, together with cytoskeleton proteins, chaperones and chaperonins were the most over-represented class of proteins identified in the infected pulldown sample (Fig. 7B), with HSPA8, HSPA2, CCT8 and TRAP1 representing the top hits (Suppl. Table S5). These observations thus suggested that RAB11A may play a scaffolding role during EV-A71 infection.

HSPA8 has been previously suggested to participate in EV-A71 assembly/maturation, but with no known association with RAB11 in such process [46]. HSPA2 is a paralog of HSPA8 with 85% amino acid sequence identity. Since many HSPAs have overlapping functions [47], it is likely that HSPA8 and HSPA2 may play a redundant role during EV-A71 infection. Although TRAP1 (a HSP90 paralog) has not been established as a pro-viral factor, other members of the HSP90 family have been shown to be important to the folding and maintenance of EV-A71 capsid proteins, by preventing their degradation [48–50]. On the other hand, involvement of chaperonins during EV-A71 infection has never been reported and was therefore further investigated. PLA signals were detected in both infected and uninfected cells (Suppl. Fig. S11), validating the physical interaction between CCT8 and RAB11A. Furthermore, siCCT8 KD led to approx. 1 log_10_ of viral titer reduction (Fig. 7C), thus supporting a pro-viral role for this chaperone. In addition, although less pronounced than in siRAB11-treated cells, higher VP0:VP2 ratio was observed in siCCT8 KD samples, supporting a role for this chaperone in virus assembly/maturation (Fig. 7D, E).

Interestingly, PLA signal was detected when probing for CCT8 and structural proteins VP1 or VP2, but not with non-structural components 3C, 3D and dsRNA (Fig. 7G). This finding supported a mechanistic model where CCT8 may be involved in folding EV-A71 structural proteins to facilitate the assembly of provirion particles and/or induce conformational changes in the provirion essential for VP0 cleavage.

Together, data from the mass spec approach and downstream experiments indicated that during EV-A71 infection, RAB11A interactions with host factors were extensively redirected towards chaperones and cytoskeleton proteins, and away from proteins involved in transport and membrane trafficking. This observation further supported that RAB11A played a minimal role in trafficking activities during infection but rather acts as an adapter or scaffold protein.

## Discussion

Previous work has shown that host factors and pathways involved in membrane trafficking are exploited by enteroviruses to support various stages of their infection cycle. For example, RAB34 and RAB17 have been reported to contribute to coxsackievirus B entry step [51]. Mannose 6-phosphate receptors (MPRs), which are transmembrane glycoproteins that target enzymes to lysosomes, have been shown to be involved in EV-A71 uncoating [44]. SNARE proteins that drive membrane fusion and cargo exchange [52], were found to participate in EV-D68 genome replication as well as in virus exit [53]. SNARE SNAP29 has also been established as a pro-viral factor during EV-A71 infection [54]. To further our understanding on how EV-A71 harnesses membrane-associated pathways and factors, we screened a siRNA library comprising of 112 genes that are associated with membrane trafficking events. RAB11A was identified as a bona fide pro-viral factor in murine and human cell lines of neuronal and muscle origin. Our data strongly supported that RAB11A and RAB11B were used interchangeably by the virus. In addition, the role of these RAB11 proteins during infection was found to be conserved across EV-A71 sub-genogroups and CVA16, another major causative agent of HFMD.

A previous study reported the screening of a similar siRNA library in human intestinal Caco-2 cells during EV-A71 infection [55]. However, neither RAB11A nor RAB11B were identified as pro-viral factors with no significant reduction in viral titers in cells treated with siRAB11A and siRAB11B respectively [55]. The discrepancy between this earlier study and our work is likely attributable to the difference in cell line that was employed for the screening. Indeed, we showed that in human RD and SH-SY5Y cell lines, simultaneous siRNA KD of both RAB11A and RAB11B was necessary to observe viral titer reduction, due to functional redundancy between both proteins. This was not true in murine NSC34 cells where siRAB11A KD alone was sufficient to observe viral titer reduction, although combined KD of RAB11A and RAB11B led to greater viral titer reduction. It is therefore very likely that combined KD of both RAB11 isoforms in Caco-2 cells would be required to observe significant reduction of the viral titers. The different outcomes following RAB11A KD in various cell lines may stem from the differential relative expression of both isoforms in those cells, which has remained unexplored to date.

Other RNA viruses including influenza and Ebola have been reported to exploit RAB11 proteins during their infection cycle [19, 21, 23]. Briefly, influenza virus was reported to exploit RAB11 to transport its ribonucleoproteins towards the plasma membrane where assembly and budding of newly formed viral particles occur [19, 21]. Similarly, during Ebola infection, RAB11 was shown to be involved in the transport of viral matrix protein VP40 towards the plasma membrane, for the release of newly made virus particles [23]. Current understanding of a possible role for RAB11 during enterovirus infection involves its interaction with viral protein 3A, and host proteins PI4KB and ACBD3, which were shown to participate to the re-programming of cholesterol shuttling processes during Poliovirus and Coxsackievirus B3 infection [25–29]. The authors proposed that those events allowed increase the intracellular pool of free cholesterol required for extensive membrane remodeling as part of the formation of replication organelles (RO), where major viral processes occur including viral RNA synthesis, viral protein translation, and virus assembly and maturation. Consistently, a Super-Resolution 3D-SIM imaging approach showed that cholesterol from plasma membranes was re-distributed to RAB11-containing recycling endosomes that were re-directed to RO during CVB3 infection [25]. Therefore, the authors of those studies proposed a role for RAB11 in the transport of free cholesterol to RO during enterovirus infection.

Our work instead did not support a role for RAB11 in trafficking activities and membrane movements during EV-A71 infection. Several lines of experimental evidence supported this conclusion. Firstly, over-expression of dominant negative or constitutively active RAB11A mutants, which lock the protein in GDP- and GTP-bound state respectively, did not affect viral replication, strongly suggesting that RAB11 GTPase activity is dispensable during EV-A71 infection, while it is an absolute requirement for RAB11 involvement in the movement of recycling endosomes [16, 17]. Secondly, our confocal multiplex imaging approach indicated that RAB11 proteins interacted with all the viral components simultaneously and as early as 6 h.p.i. with no evidence of time-dependent interactions, which would have been expected should RAB11 be involved in transporting viral components across various subcellular locations. Further analysis of subcellular compartment markers indicated that those interactions likely occurred at the RO.

Furthermore, we showed that RAB11 was not involved in the virus entry steps (receptor-mediated endocytosis and viral uncoating), nor in viral protein translation and RNA genome replication. In contrast, our data supported that RAB11 may be involved in the virus assembly and maturation process as evidenced by higher VP0:VP2 ratio in cells treated with siRAB11. Together, these findings led us to propose that RAB11 may act as a scaffold or adapter protein at the RO for optimal assembly and/or VP0 cleavage-mediated maturation of newly formed viral particles. The identification of chaperones as top interacting partners of RAB11A during EV-A71 infection supports this view.

One possible explanation for the discrepancy between our study and earlier work describing the role of RAB11 in cholesterol shuttling processes during enterovirus infection [25] could be that while RAB11 is found on cholesterol-loaded recycling endosomes, it actually does not contribute to the movement of those recycling endosomes towards the RO. Instead, this activity may be performed by other host factors. Alternatively, it is also possible that poliovirus and CVB3 exploit RAB11 proteins according to a different mechanism compared to EV-A71.

**Fig. 8:**
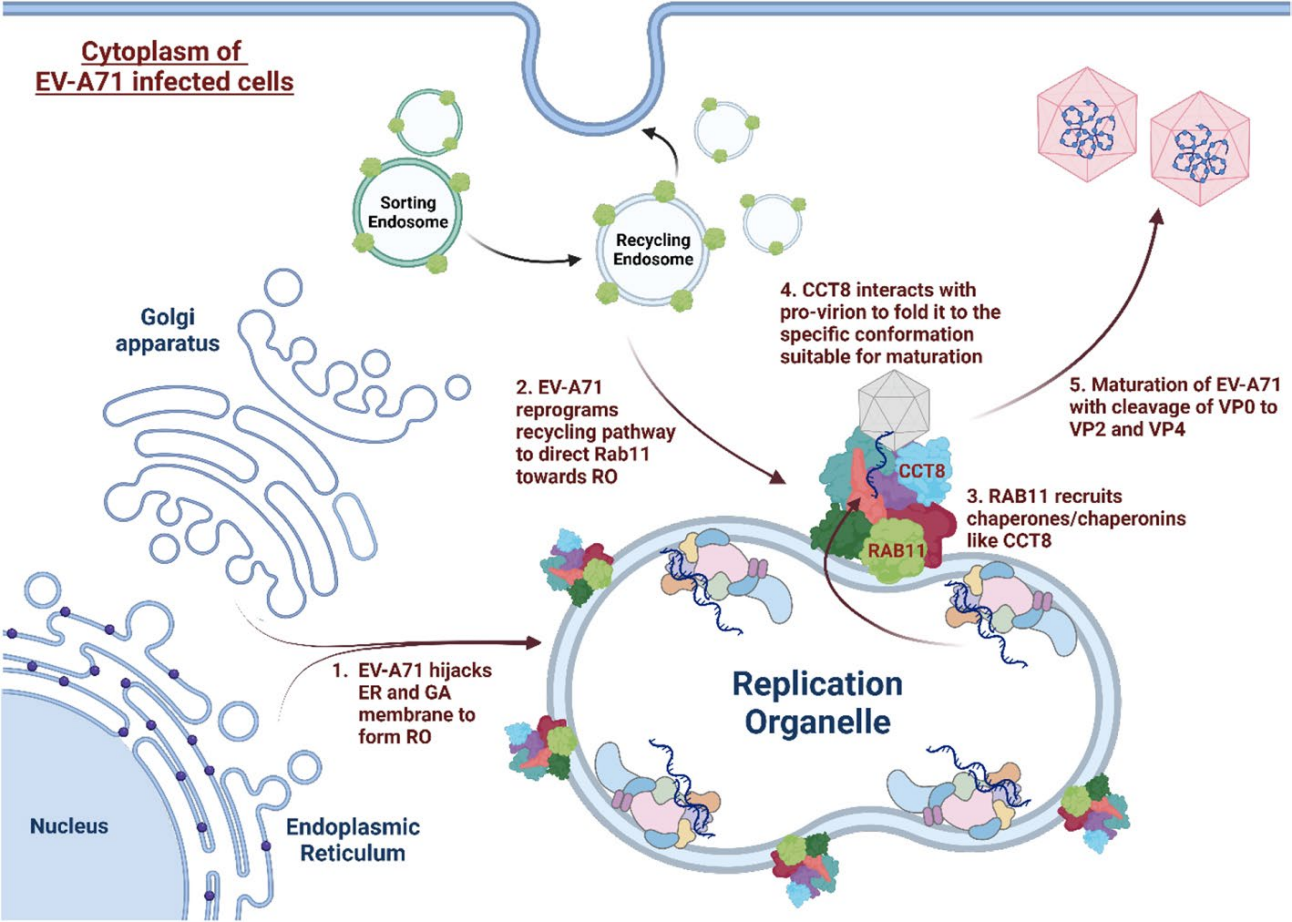
A working model of RAB11 involvement in EV-A71 infection cycle. EV-A71 induces host membrane rearrangement for the formation of replication organelles (ROs) during infection. Such events involve hijacking the membranes from ER, GA and recycling endosomes. During the process, RAB11 is re-routed to the ROs, where it then interacts with  viral proteins and recruits host factors essential for virus assembly and maturation . An important class of host factors recruited by RAB11 are chaperones/chaperonins like CCT8 that likely participate in (i) inducing a structural change to the individual viral capsid subunits permitting the assembly of procapsids or (ii) inducing a conformational change in the provirion particle that enables the cleavage of VP0 to VP2 and VP4 during the final maturation step of EV-A71

While little is known about the assembly and maturation processes of EV-A71 virus, our work seems to implicate RAB11 involvement. Our model proposes that through re-directing of recycling endosomes to RO, RAB11 proteins recruit a variety of chaperone proteins that together participate in the assembly and folding of newly formed virions, enabling VP0 cleavage (Fig. 8). A recent report has described the involvement of HSPAs at all stages of EV-A71 infection cycle, with HSPA8 and HSPA9 specifically involved in the virus maturation step [46]. Consistently, we identified HSPA8 as one of the top interacting partners of RAB11A during EV-A71 infection, along with HSPA1 and HSPA2. More interestingly and uniquely, we identified RAB11A-interacting chaperone CCT8 as a *bona fide* pro-viral factor during EV-A71 infection. CCT8 is a component of chaperone complex TRiC/CCT, which plays a critical role in the folding of cytoskeleton proteins such as tubulin and actin [56]. TRiC consists of 8 CCT subunits that bind to different targets and substrates, while monomeric CCT subunits are functionally active too [56–59]. CCTs were found to be exploited by a number of viruses including reovirus and HCV for folding and stabilizing their viral protein structure [60, 61]. A previous transcriptomic and proteomic study reported that CCT8 gene expression and protein levels were reduced in EV-A71 infected RD cells and the authors speculated that such reduction could favor cytoskeleton disruption and re-organization to facilitate formation of RO [62]. Here, we propose that CCT8 monomers and/or TRiC may either be involved in folding individual viral structural proteins or inducing conformational changes in newly formed EV-A71 provirions to facilitate VP0 cleavage.

## Conclusion

This work contributes further to our fundamental knowledge and understanding of the dynamic and complex molecular interactions between EV-A71 and its mammalian host. Given the lack of effective antiviral treatment to fight this disease, the identification of novel host factors exploited by EV-A71 represents a potential avenue for developing host directed therapies.

### Supplementary Information

Supplementary Material 1.

## Data Availability

Raw data and materials described in this paper is available from corresponding author upon request.

## Abbreviations

CO2: Carbon dioxide
Co-IP: Co-immunoprecipitation
CVA16: Coxsackievirus A16
DAPI: 4’, 6-Diamidino-2-phenylindole
DMEM: Dulbecco’s Modified Eagle’s Medium
DN: Dominant negative
DPEC: Diethylpyrocarbonate
dsRNA: Double stranded RNA
ER: Endoplasmic reticulum
EV-A71: Enterovirus-A71
EV-D68: Enterovirus-D68
FACS: Fluorescence-activated cell sorting
FBS: Fetal bovine serum
GA: Golgi apparatus
GFP: Green fluorescent protein
HFMD: Hand, Foot and Mouth Disease
HSPA: Heat shock protein 70 family A
IFA: Immunofluorescence assay
IRES: Internal ribosome entry site
KD: Knockdown
MPR: Mannose 6-phosphate receptor
MOI: Multiplicity of infection
NTC: Non-targeting control
PBS: Phosphate-buffered saline
PCC: Pearson correlation coefficient
PHB: Prohibitin
PLA: Proximity ligation assay
qPCR: Quantitative polymerase chain reaction
RNA: Ribonucleic acid
RO: Replication organelle
RT: Room temperature
siRNA: Small interfering RNA
SNARE: Soluble N-ethylmaleimide-sensitive factor attachment protein receptor
TGN: Trans-Golgi network
WT: Wild type
