# Supplementary material for: Enterovirus-A71 exploits RAB11 to recruit chaperones for virus morphogenesis
Source: J Biomed Sci. 2024 Jun 28;31:65. doi: 10.1186/s12929-024-01053-2 (PMC11212238; doi:10.1186/s12929-024-01053-2)
Supplement: Supplementary file 1 — Supplementary Material 1. [file 12929_2024_1053_MOESM1_ESM.pdf]

Supplemental Tables and Figures

Table S1. siRNA species employed in this study.

Gene Target	Concentration Used	siRNA Cat #	siRNA name	Sequences
Mus musculus RAB11A	50nM	J-040863-09	siRAB11A #1	GUACAGGGCUAUAACGUCU
		J-040863-10	siRAB11A #2	UAAGAGUGAUUUACGUCAU
		J-040863-11	siRAB11A #3	GCGACGACGAGUACGACUA
		J-040863-12	siRAB11A #4	UAACAGAGAUAUACCGCAU
Homo sapiens RAB11A	25nM	J-004726-07	siRAB11A #7	GCAACAAUGUGGUUCCUAU
		J-004726-08	siRAB11A #8	CAAGAGCGAUAUCGAGCUA
		J-004726-09	siRAB11A #9	GUGCAGUGCUGUCAGAACA
		J-004726-10	siRAB11A #10	GAGAUUUACCGCAUUGUUU
Homo sapiens RAB11B	25nM	J-004727-06	siRAB11B #6	UAACGUAGAGGAAGCAUUC
		J-004727-07	siRAB11B #7	GAGUACGACUACCUAUUCA
		J-004727-08	siRAB11B #8	UCGCCAAGCACCUGACCUA
		J-004727-09	siRAB11B #9	CAACUUGUCCUUCAUCGAG
Homo sapiens CCT8	25nM	J-008566-06	siCCT8 #6	UGAAUGGCCCUUCGUGAUA
		J-008566-07	siCCT8 #7	GCACAGGUAAGGCUAACAA
		J-008566-08	siCCT8 #8	GAGAUAGACUCCCUGUUCG
		J-008566-09	siCCT8 #9	GAGGAGAGGACUUCGACAA
Mus musculus ROCK2	50nM	J-040429-05	siROCK2 #5	GAGAUUACCUUACGGAAAA
		J-040429-06	siROCK2 #6	GGACAUGAGUUUAUUCCUA
		J-040429-07	siROCK2 #7	GCAAUGAAGCUUCUUAGUA
		J-040429-08	siROCK2 #8	CACAACAGAUGAUCAAAUA
Mus musculus LIMK1	50nM	J-043923-05	siLIMK1 #5	GAUCAAUGGUCGUAGUUAU
		J-043923-06	siLIMK1 #6	GCGAGGUGAUGGUGAUGAA
		J-043923-07	siLIMK1 #7	UAACUCCAGUCAUCGAACA
		J-043923-08	siLIMK1 #8	CCAAAGGGCUGGUCAUGGU
Mus musculus CBLB	50nM	J-051830-05	siCBLB #5	GAGCAUACUUCGAGAAUUU
		J-051830-06	siCBLB #6	GCACCAAGCCUGGAAGUUA
		J-051830-07	siCBLB #7	AGUAUGAACUGUAUUGUGA
		J-051830-08	siCBLB #8	CCUGUUCGGUCUUGUGAUA
Non-targeting Control Pool	Varied	D-001810-10	siNTC	UGGUUUACAUGUCGACUAA
				UGGUUUACAUGUUGUGUGA
				UGGUUUACAUGUUUUCUGA
				UGGUUUACAUGUUUUCCUA

Table S2. Primary and secondary antibodies used in Western blot analyses.

Antibody	Specie	Dilution factor	Cat #
Anti-RAB11A	Rabbit	1:1000	Abcam; ab128913
Anti-RAB11B	Rabbit	1:1000	Abcam; ab175925
Anti-CCT8	Rabbit	1:1000	Atlas Antibodies; HPA029426
Anti-EVA71 VP1	Mouse	1:1000	Abnova; MAB1255-M08
Anti-EVA71 VP2	Mouse	1:1000	Merck Millipore; MAB979
Anti-EVA71 3C	Mouse	1:1000	Genetex; GTX630193
Anti-EVA71 3C	Rabbit	1:1000	Genetex; GTX132357
Anti-EVA71 3D	Mouse	1:1000	Genetex; GTX630191
Beta-Actin	Mouse	1:1000	Invitrogen; MA5-15739
Beta-Actin	Rabbit	1:10000	Abcam; ab8227
Anti-Rabbit IgG (H + L)-HRP Conjugate	Goat	1:3000	Bio-Rad; 1706515
Anti-Mouse IgG (H + L)-HRP Conjugate	Goat	1:3000	Bio-Rad; 1706516

Table S3. Sequences of primers used in Real-time polymerase chain reaction (qPCR)

Set No.	Primer Name	Sequence	Ta
1	EV-A71 VP1 Forward	GCACAGGTCTCAGTTCCGTT	58°C
	EV-A71 VP1 Reverse	CACGCCTGACATGCTTCAT	
2	RAB11A Forward	TGGAGATTCTGGTGTTGGAAAG	58°C
	RAB11A Reverse	ACCTGGATGCTTCTTGTTGC	
3	RAB11B Forward	GGCAACAAGAGTGACCTGC	58°C
	RAB11B Reverse	TGGAATCCAAGGCTGAGGTC	
4	GAPDH Forward	GAGTCAACGGATTTGGTCGT	60°C
	GAPDH Reverse	TTGATTTTGGAGGGATCTCG	

Table S4. Antibodies used for IFA and PLA.

Antibody	Type	Species	Dilution factor	Cat #
Anti-RAB11A	Primary	Rabbit	1:250	Abcam; ab128913
Anti-RAB11B	Primary	Rabbit	1:250	Abcam; ab175925
Anti-CCT8	Primary	Rabbit	1:50	Atlas Antibodies; HPA029426
Anti-CCT8	Primary	Mouse	1:750	Proteintech; 67539-1-Ig
Anti-Calreticulin (AF647)	Primary; Conjugated	Rabbit	1:100	Abcam; ab196159
Anti-GM130 (AF647)	Primary; Conjugated	Rabbit	1:100	Abcam; ab195303
Anti-Transferrin Receptor (AF647)	Primary; Conjugated	Rabbit	1:100	Abcam; ab187777
Anti-Calreticulin (AF488)	Primary; Conjugated	Rabbit	1:100	Abcam; ab196158
Anti-GM130 (AF594)	Primary; Conjugated	Rabbit	1:100	Abcam; ab277236
IgG isotype control	Primary	Mouse	1:750	Proteintech; 66360-2-Ig
Alexa Fluor® Plus 405 goat anti-mouse IgG	Secondary	Goat	1:500	Invitrogen; A48258
Alexa Fluor® 488 goat anti-mouse IgG	Secondary	Goat	1:500	Invitrogen; A-11001
Alexa Fluor® 594 goat anti-rabbit IgG	Secondary	Goat	1:500	Invitrogen; A-11012
Anti-EVA71 VP1	Primary	Mouse	1:500	Abnova; MAB1255-M08
Anti-EVA71 VP2	Primary	Mouse	1:500	Merck Millipore; MAB979
Anti-EVA71 3C	Primary	Mouse	1:250	Genetex; GTX630193
Anti-EVA71 3D	Primary	Mouse	1:250	Genetex; GTX630191
Anti-dsRNA	Primary	Mouse	1:250	Scicons; J2

Table S5. Chaperone/chaperonin hits identified in infected pulldown samples with enrichment compared to uninfected samples.

No.	Protein	Protein Class	Fold change (Infected/Uninfected)
**1**	HSPA8	Chaperone	8.52
**2**	HSPA2	Chaperone	8.52
**3**	CCT8	Chaperonin	4.26
**4**	TRAP1	Chaperone	4.26
**5**	HSPA90AB1	Chaperone	2.09
**6**	CCT3	Chaperonin	1.99
**7**	HSP90AA1	Chaperone	1.95
**8**	HSPA1A	Chaperone	1.75
**9**	HSPA1B	Chaperone	1.75

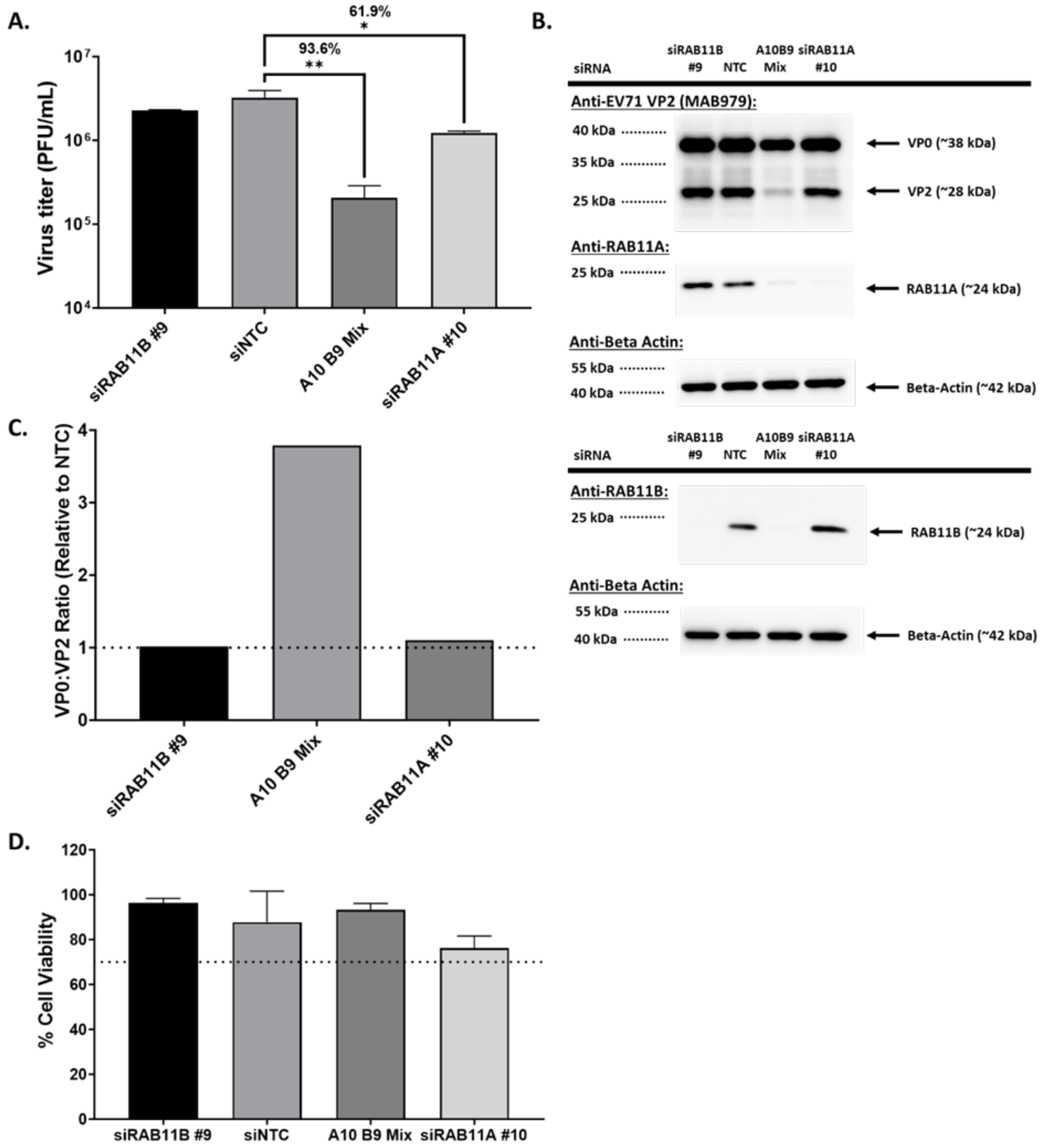

Figure S1. Effects of RAB11A and RAB11B siRNA KD in SH-SY5Y cells.

SH-SY5Y cells were transfected with deconvoluted human siRAB11A #10, siRAB11B #9, a mix of both or siNTC, followed by infection with EV-A71 S41 (MOI 0.1) at 48h.p.t. At 24h.p.i, the culture supernatants and cells were harvested. **(A)** Virus titers in the cultured supernatants were determined by plaque assay. Statistical analysis was performed using Kruskal-Wallis test against siNTC treatment (*p<0.05, **p<0.01, ***p<0.001, ****p<0.0001). The percentage of viral titer reduction was indicated above the asterisk. **(B)** The cell lysates were subjected to Western blot analysis using anti-VP2, anti-RAB11A, anti-RAB11B and anti B-actin antibodies. **(C)** VP0:VP2 ratio relative to NTC, based on signal band intensity measured by ImageLab. **(D)** AlamarBlue assay was performed on uninfected siRNA-treated cells. The readout was relative to non-siRNA-treated cells to determine the percentage of cell viability. Percentage above 70% (indicated by the dotted line) was considered non-cytotoxic.

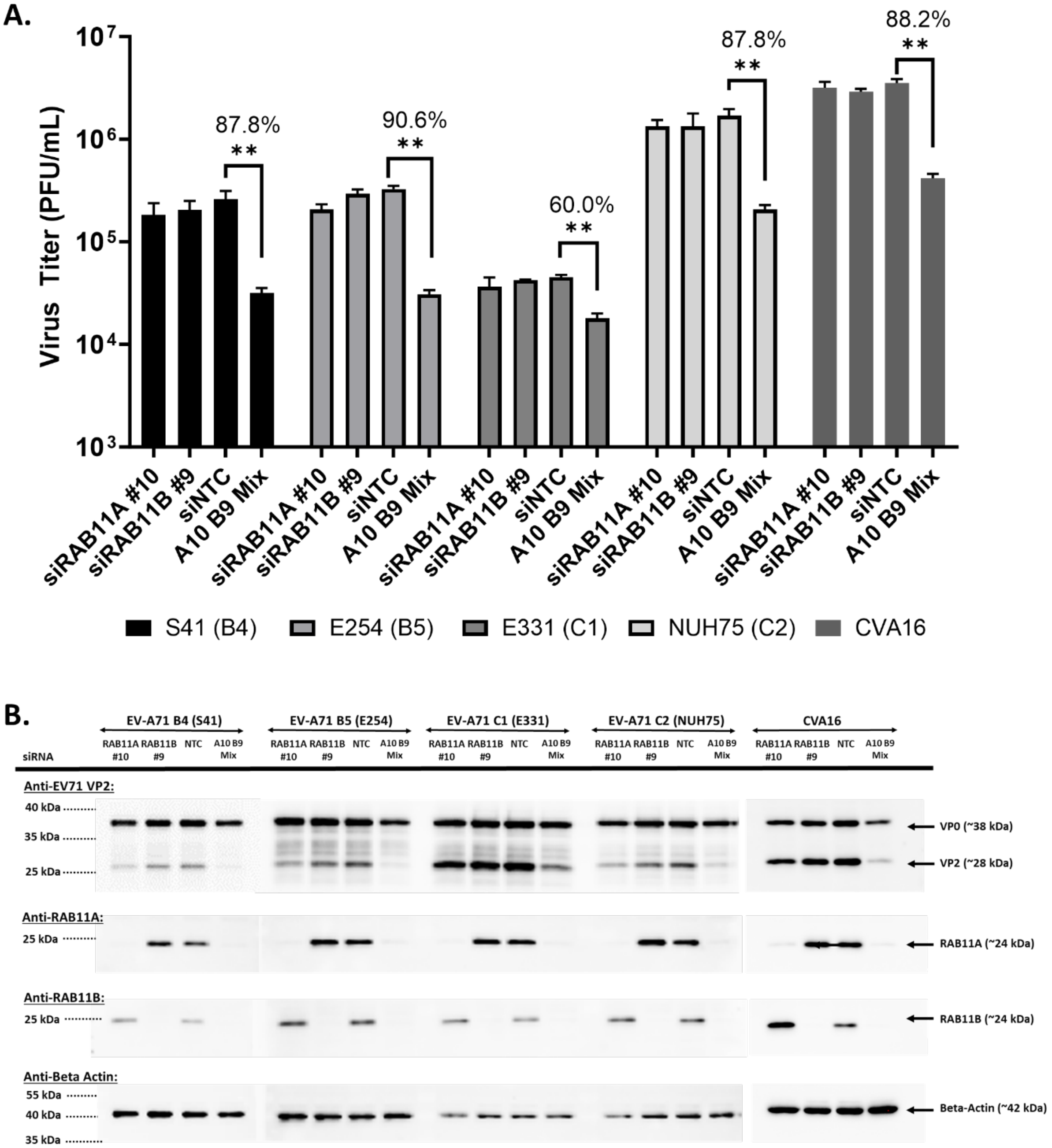
A.
Virus Titer (PFU/mL)
87.8%
90.6%
60.0%
87.8%
88.2%
siRAB11A #10
siRAB11B #9
siNTC
A10 B9 Mix
S41 (B4)
E254 (B5)
E331 (C1)
NUH75 (C2)
CVA16
B.
EV-A71 B4 (S41)
EV-A71 B5 (E254)
EV-A71 C1 (E331)
EV-A71 C2 (NUH75)
CVA16
siRNA
RAB11A #10
RAB11B #9
NTC
A10 B9 Mix
Anti-EV71 VP2:
Anti-RAB11A:
Anti-RAB11B:
Anti-Beta Actin:
VP0 (~38 kDa)
VP2 (~28 kDa)
RAB11A (~24 kDa)
RAB11B (~24 kDa)
Beta-Actin (~42 kDa)

C.

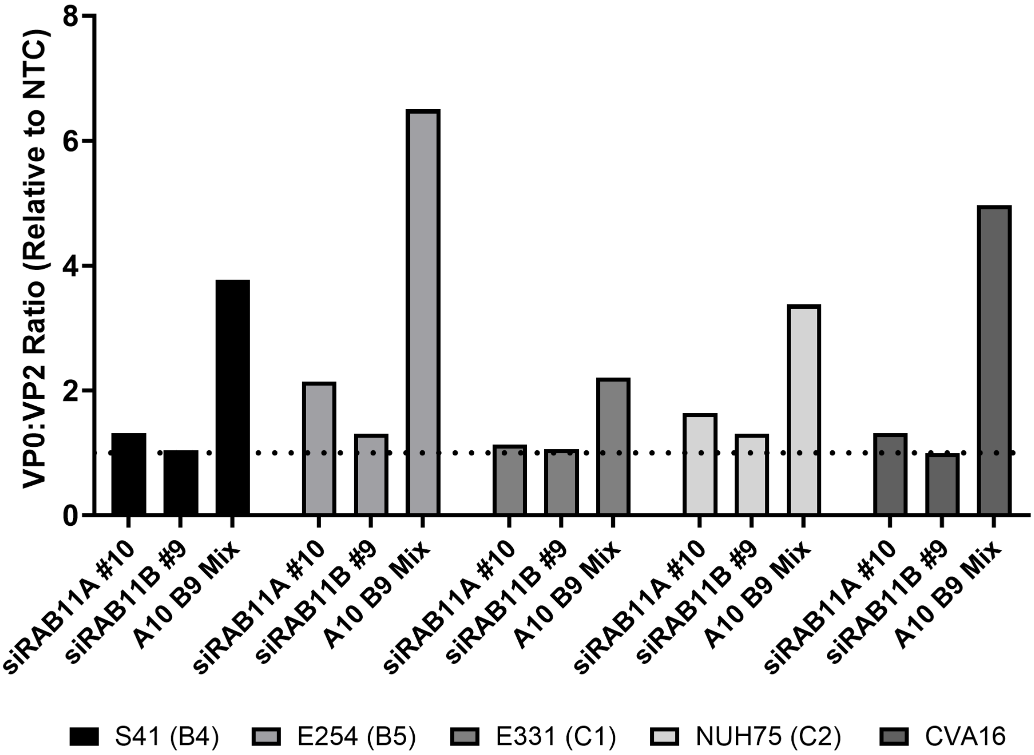

Figure S2: Effect of RAB11A and RAB11B siRNA KD in SHSY5Y cells infected with various EV-A71 subgenotypes and CVA16.

SHSY5Y cells were transfected with siRAB11A#10, siRAB11B#9, a mix of both (A10B9) or siRNA NTC, followed by infection with various EV-A71 subgenotypes and CVA16. The culture supernatants and cells were collected at 24 h.p.i. **(A)** The virus titers in culture supernatants were determined by plaque assays. Statistical analysis was performed using Kruskal-Wallis test against siNTC treatment (*p<0.05, **p<0.01, ***p<0.001, ****p<0.0001). The percentage of viral titer reduction was indicated above the asterisk. **(B)** The cell lysates were analysed by Western blot using anti-VP2, anti-RAB11A, and anti-RAB11B antibodies. **(C)** VP0:VP2 ratio relative to NTC, based on signal band intensity measured by ImageLab.

A.

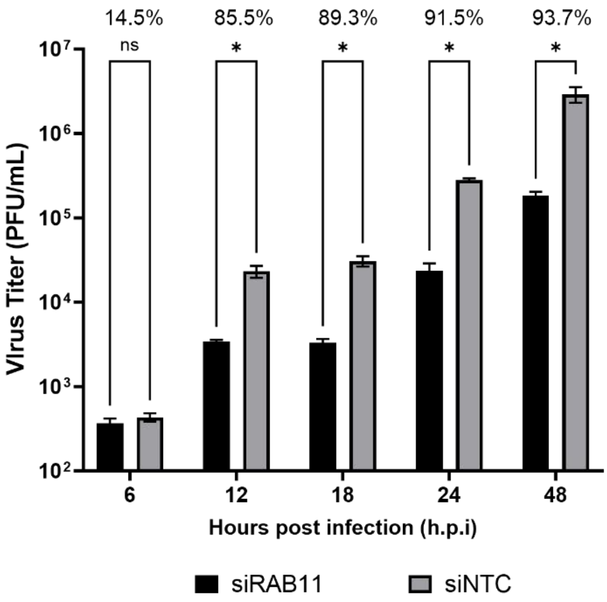

14.5%
85.5%
89.3%
91.5%
93.7%
ns
*
Virus Titer (PFU/mL)
10^7
10^6
10^5
10^4
10^3
10^2
6
12
18
24
48
Hours post infection (h.p.i)
siRAB11
siNTC

B.

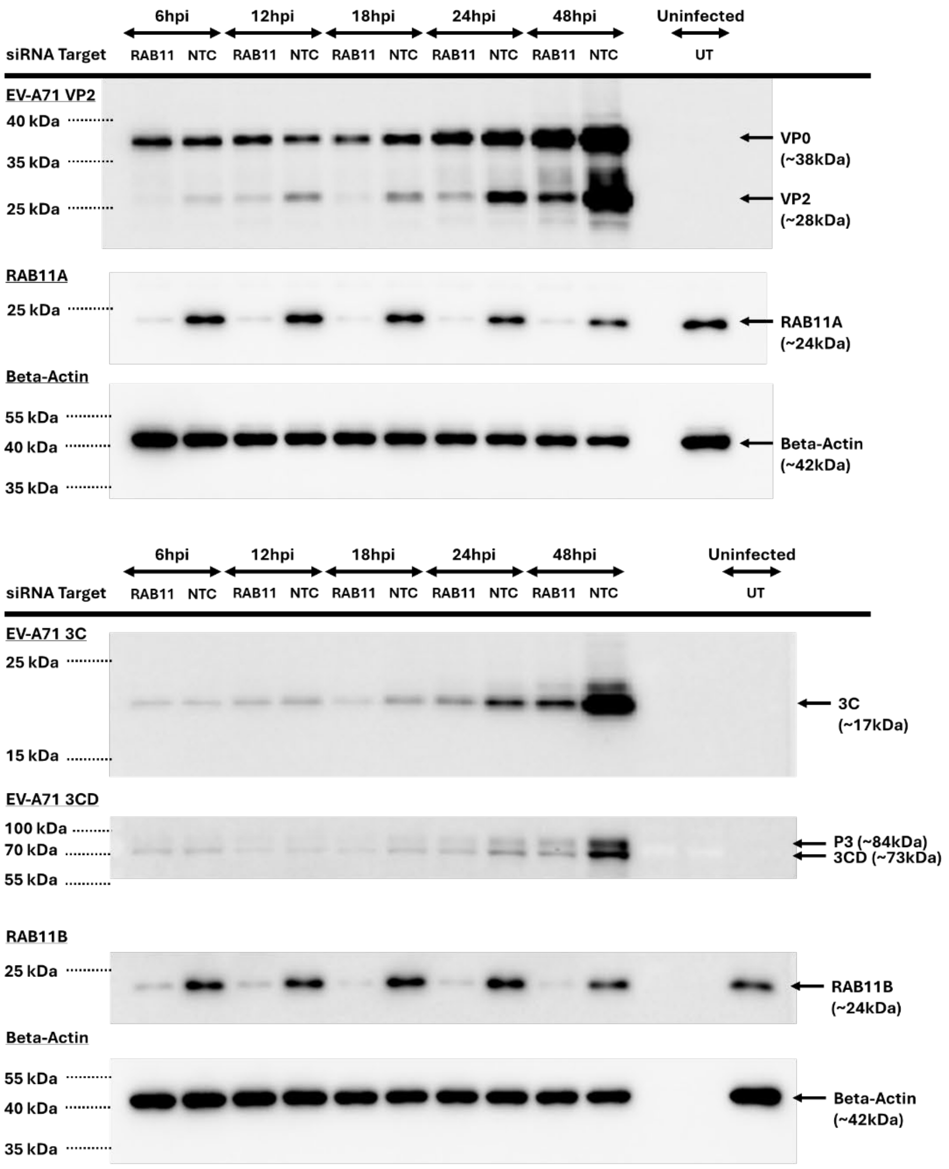

6hpi
12hpi
18hpi
24hpi
48hpi
Uninfected
siRNA Target
RAB11
NTC
UT
EV-A71 VP2
40 kDa
35 kDa
25 kDa
VP0 (~38kDa)
VP2 (~28kDa)
RAB11A
25 kDa
RAB11A (~24kDa)
Beta-Actin
55 kDa
40 kDa
35 kDa
Beta-Actin (~42kDa)
EV-A71 3C
25 kDa
15 kDa
3C (~17kDa)
EV-A71 3CD
100 kDa
70 kDa
55 kDa
P3 (~84kDa)
3CD (~73kDa)
RAB11B
25 kDa
RAB11B (~24kDa)
Beta-Actin
55 kDa
40 kDa
35 kDa
Beta-Actin (~42kDa)

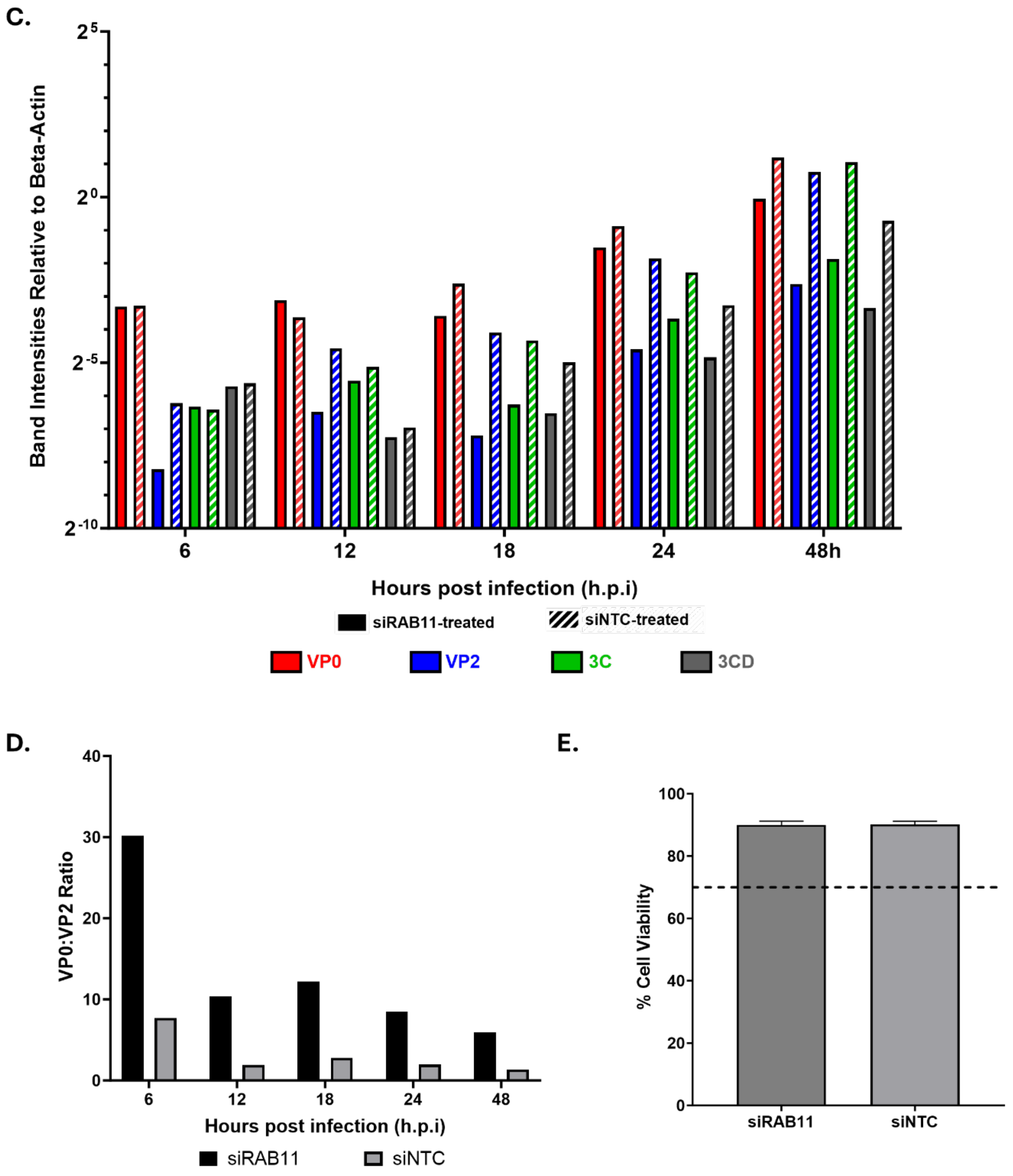

Figure S3: EV-A71 viral kinetic profile in siRAB11-treated cells.

SH-SY5Y cells were transfected with siRAB11 or with siNTC, followed by infection with EV-A71 S41 (MOI 0.1) at 48h.p.t. The culture supernatants and cell lysates were harvested at various timepoints ranging from 6h.p.i to 48h.p.i. **(A)** Virus titers in the culture supernatants were determined by plaque assay. Statistical analysis was performed using Mann-Whitney U test against siNTC treatment (*p<0.05, **p<0.01, ***p<0.001, ****p<0.0001). The percentage of virus titer reduction in siRab11-treated samples relative to siNTC samples was indicated above the asterisk. **(B)** The cell lysates were subjected to Western blot analysis using anti-VP2, anti-3C, anti-RAB11A, anti-RAB11B and anti B-actin primary antibodies. Band intensities were determined using ImageLab and were normalised to Beta-actin. **(C)** VP0, VP2, 3C and 3CD band intensities of siRAB11 and siNTC treatment were calculated. **(D)** VP2:VP0 ratio were further computed. **(E)** AlamarBlue assay was performed on uninfected siRNA-treated cells at 48 hours post-transfection. The readout for each treatment was relative to untreated cells to determine the percentage of cell viability. Percentage above 70% (indicated by the dotted line) was considered non-cytotoxic.

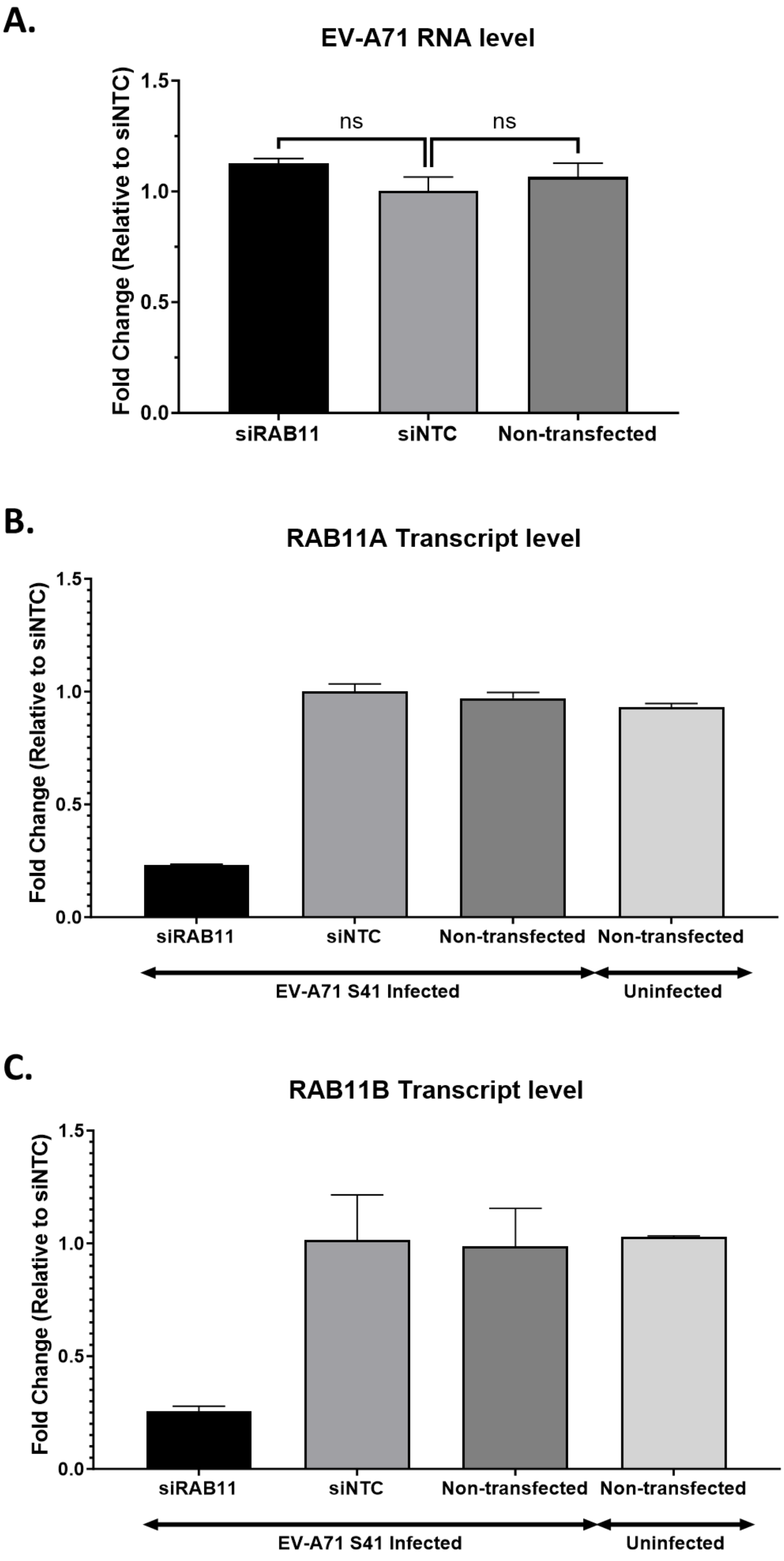

Figure S4: Effect of RAB11 KD on viral RNA at 4 hours post-EV-A71 infection.
SH-SY5Y cells were transfected with siRAB11 or siNTC, followed by infection with EV-A71 S41 (MOI 1). The cells were harvested at 4 h.p.i., lysed and total RNA was extracted. The levels of **(A)** EV-A71 RNA, **(B)** RAB11A and **(C)** RAB11B transcripts were quantified by qPCR and fold changes relative to siNTC were plotted. Statistical analysis was performed using Kruskal-Wallis test against siNTC treatment (*$p<0.05$, **$p<0.01$, ***$p<0.001$, ****$p<0.0001$).

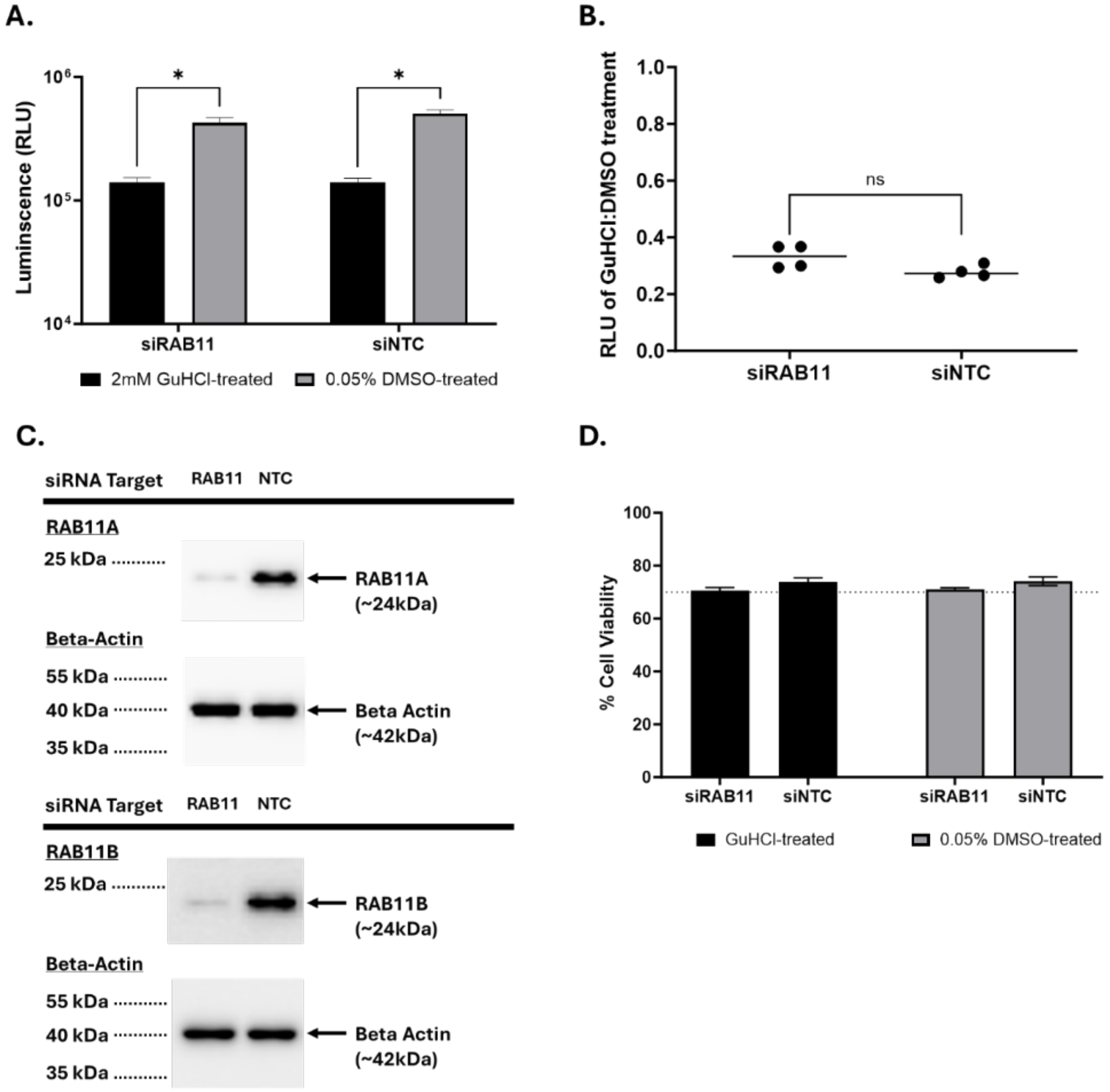

Figure S5: Effect of guanidine hydrochloride on Luciferase activity in SH-SY5Y cells. SH-SY5Y cells were treated with siRab11 or siNTC, then transfected with 500ng of *in vitro* transcribed EV71-Luc RNA. At 4 hours post-transfection, culture medium was replaced with complete growth medium containing 2mM of GuHCl or 0.05% DMSO. **(A)** Luciferase activity and **(D)** cytotoxicity were measured at 24 hours post-transfection. **(B)** The ratio of luciferase signal in GuHCl-treated samples relative to that in samples treated with 0.05% DMSO was determined. **(C)** siRab11-treated cells were also harvested to assess the siRAB11 KD efficiency by Western blot, using anti-RAB11A, anti-RAB11B and Anti-B-actin primary antibodies. Statistical analysis was performed for (A) and (B) using Mann-Whitney U test against siNTC treatment (*p<0.05, **p<0.01, ***p<0.001, ****p<0.0001).

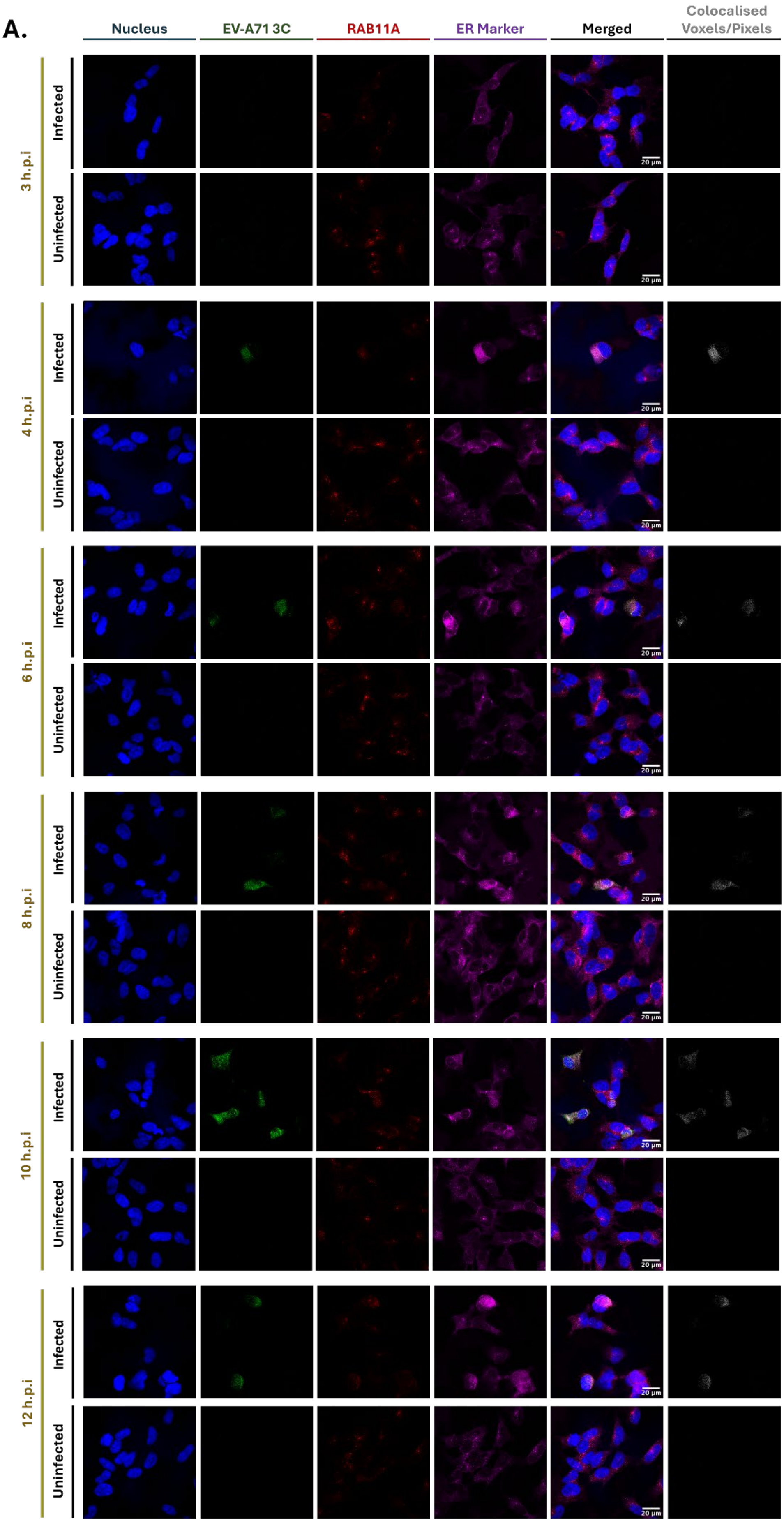
A.
Nucleus
EV-A71 3C
RAB11A
ER Marker
Merged
Colocalised Voxels/Pixels
3 h.p.i
4 h.p.i
6 h.p.i
8 h.p.i
10 h.p.i
12 h.p.i
Infected
Uninfected
20 µm

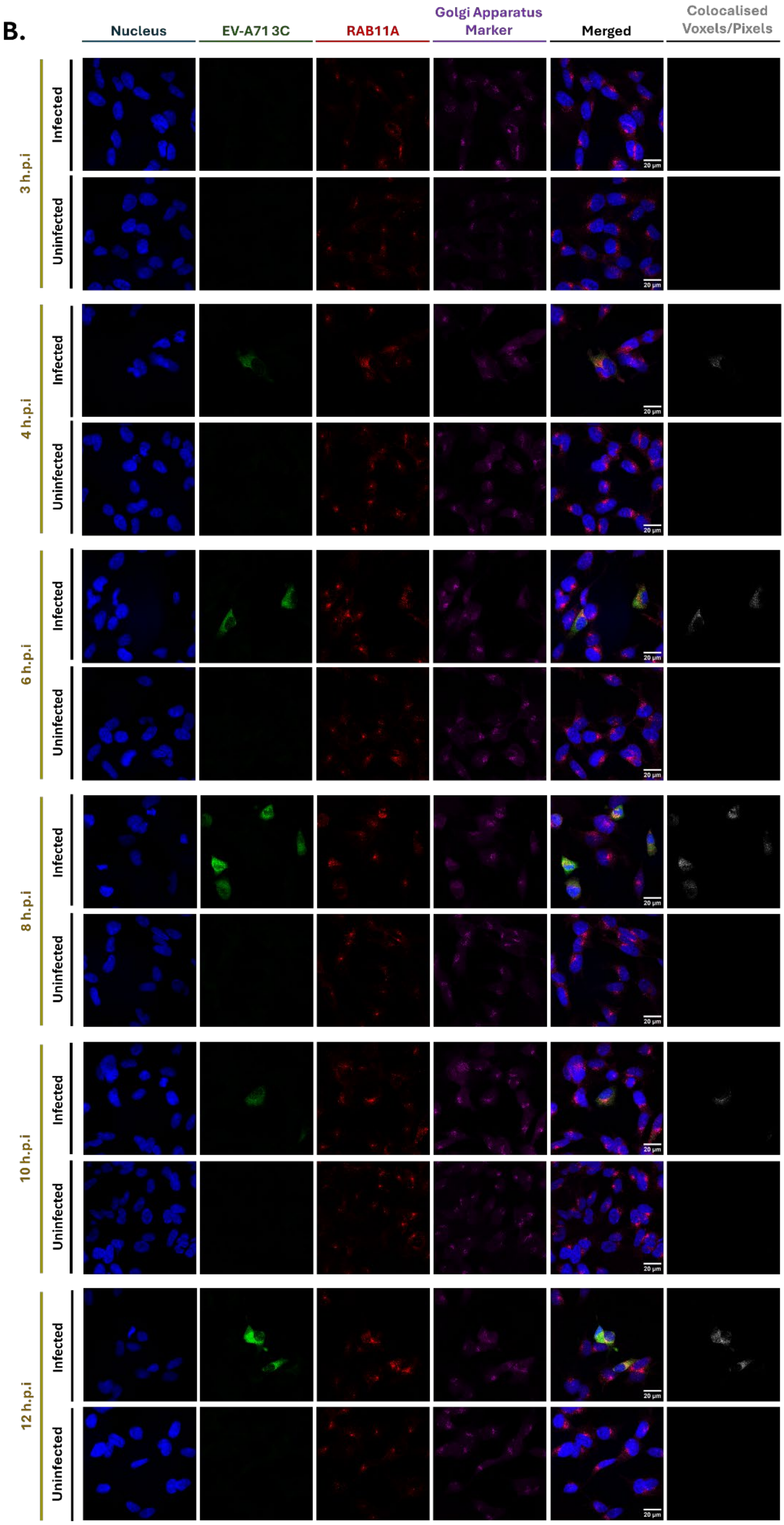

B.
Nucleus
EV-A71 3C
RAB11A
Golgi Apparatus Marker
Merged
Colocalised Voxels/Pixels
3 h.p.i
4 h.p.i
6 h.p.i
8 h.p.i
10 h.p.i
12 h.p.i
Infected
Uninfected
20 µm

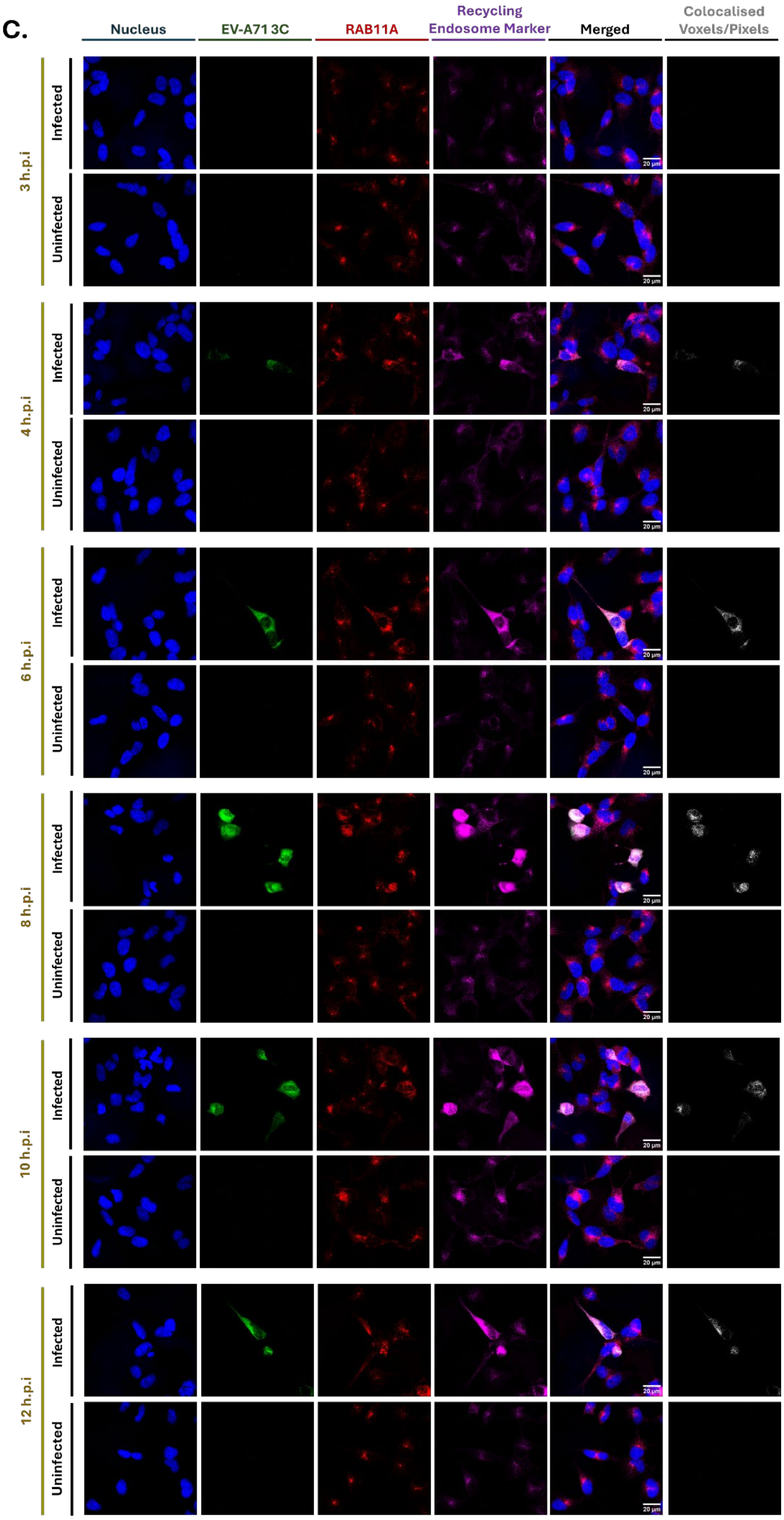
C.
Nucleus
EV-A71 3C
RAB11A
Recycling Endosome Marker
Merged
Colocalised Voxels/Pixels
3 h.p.i
4 h.p.i
6 h.p.i
8 h.p.i
10 h.p.i
12 h.p.i
Infected
Uninfected
20 µm

D.

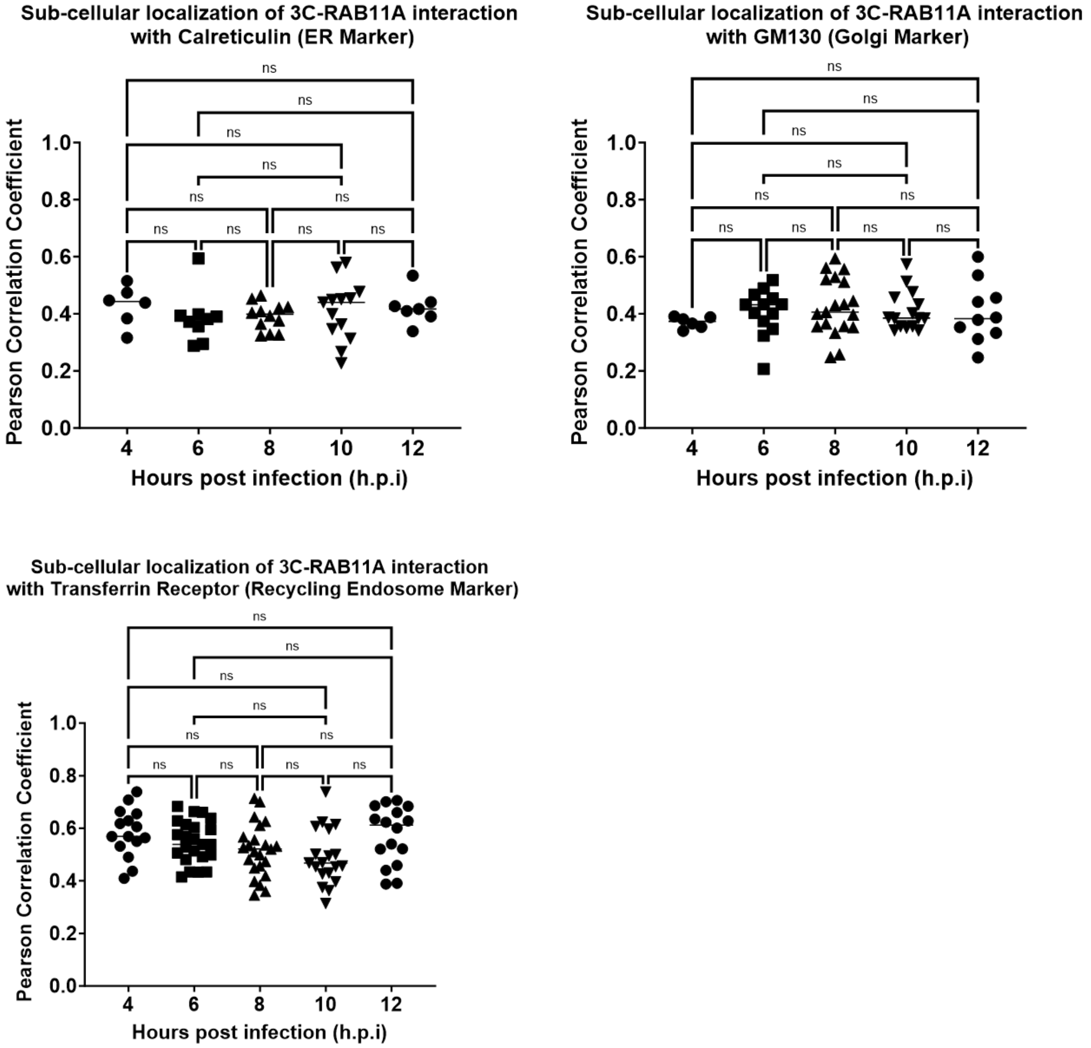

Figure S6. Interactions between RAB11A and viral protein 3C/3CD in various subcellular compartments over time.

SH-SY5Y cells were infected with EV-A71 S41 (MOI 0.1). At the indicated time points post-infection, the cells were fixed and permeabilized, followed by staining with anti-RAB11A and anti-3C primary antibodies, and labelled secondary antibodies. Cells were then further stained with antibodies specific to compartment markers calreticulin for endoplasmic reticulum **(A)**, GM130 for Golgi apparatus **(B)** and transferrin receptor (Tfr) for small recycling endosomes **(C)** prior to DAPI staining. Confocal images were captured under 100X objective and were analyzed using Fiji software to determine the voxels/pixels that represent 3 channel (green, red, magenta) co-localization. Briefly, a mask representing the co-localization of RAB11A and each viral component was delineated and subsequently overlayed with compartment marker signals to generate the 'colocalized voxels' images shown on the far right column**. (D)** Pearson correlation coefficient (PCC) values were computed using Fiji software. PCC value of 0 = no co-localization; 0.1 – 0.3 = weak co-localization; 0.3 – 0.5 = moderate co-localization; 0.5-1 = strong co-localization. Statistical analysis was performed using Kruskal-Wallis test with Dunn correction against siNTC treatment (*p<0.05, **p<0.01, ***p<0.001, ****p<0.0001).

A.

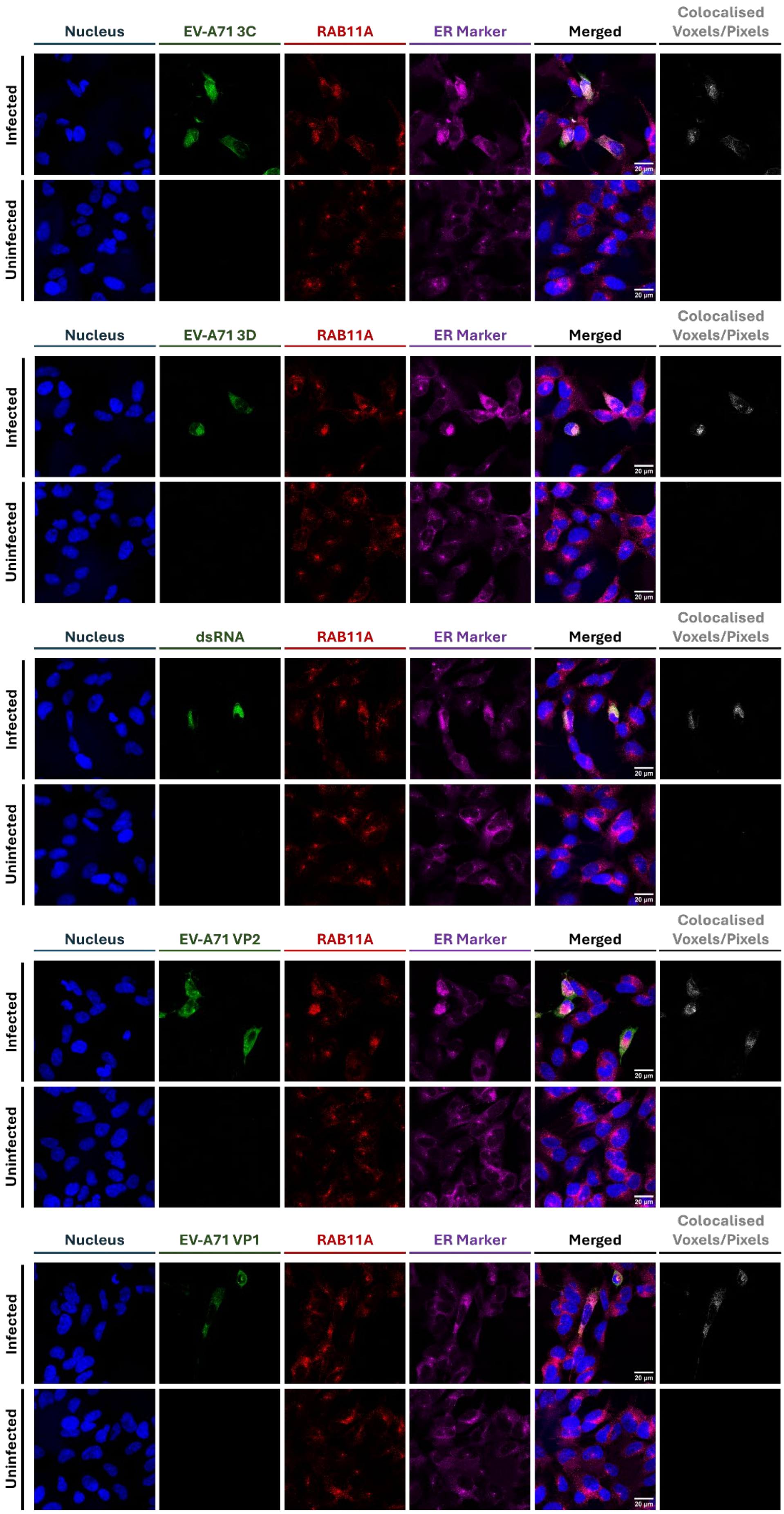
Nucleus
EV-A71 3C
RAB11A
ER Marker
Merged
Colocalised Voxels/Pixels
Infected
Uninfected
EV-A71 3D
dsRNA
EV-A71 VP2
EV-A71 VP1
20 µm

B.

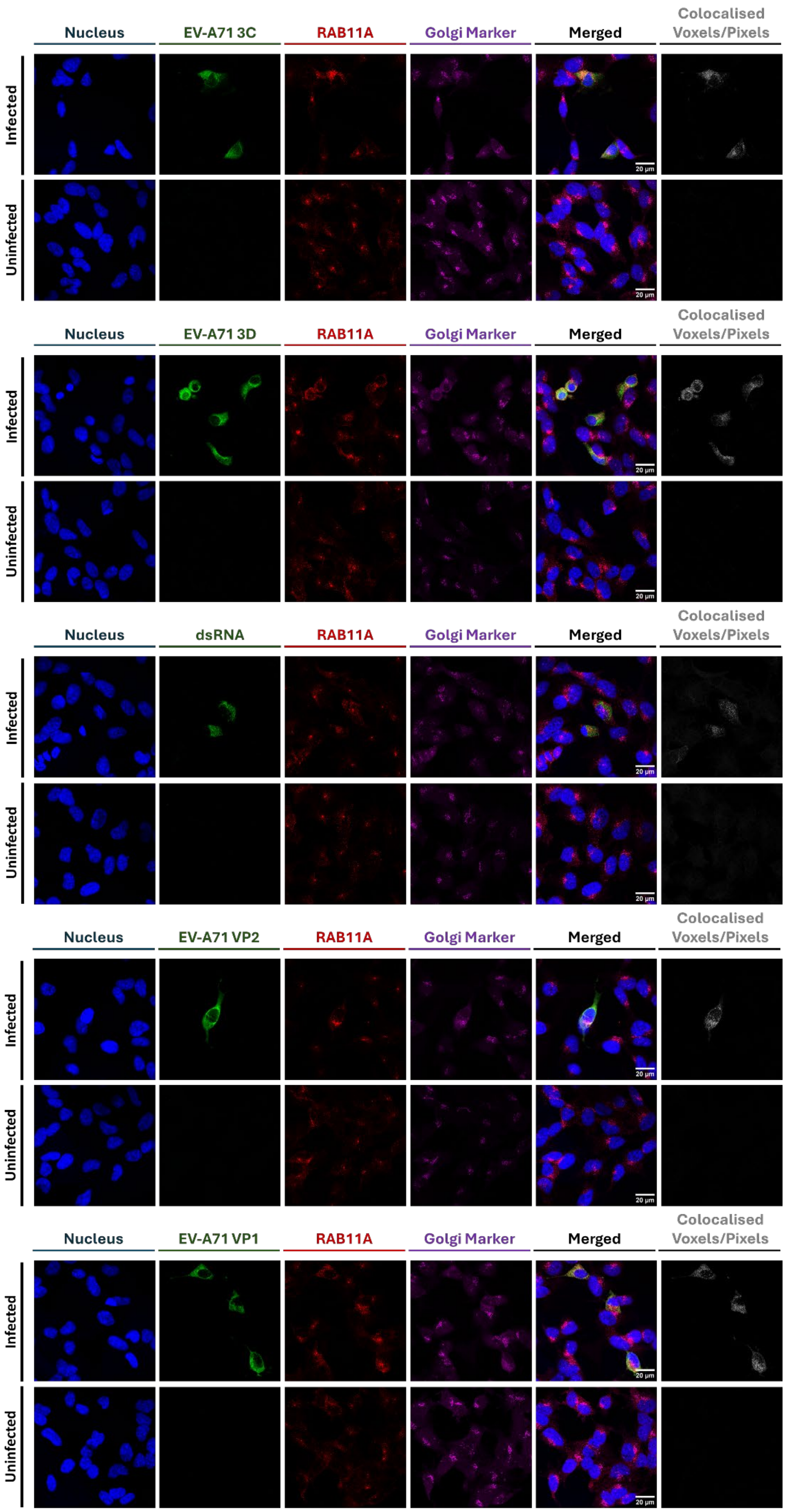

Nucleus
EV-A71 3C
RAB11A
Golgi Marker
Merged
Colocalised Voxels/Pixels
Infected
Uninfected
Nucleus
EV-A71 3D
RAB11A
Golgi Marker
Merged
Colocalised Voxels/Pixels
Infected
Uninfected
Nucleus
dsRNA
RAB11A
Golgi Marker
Merged
Colocalised Voxels/Pixels
Infected
Uninfected
Nucleus
EV-A71 VP2
RAB11A
Golgi Marker
Merged
Colocalised Voxels/Pixels
Infected
Uninfected
Nucleus
EV-A71 VP1
RAB11A
Golgi Marker
Merged
Colocalised Voxels/Pixels
Infected
Uninfected

C.

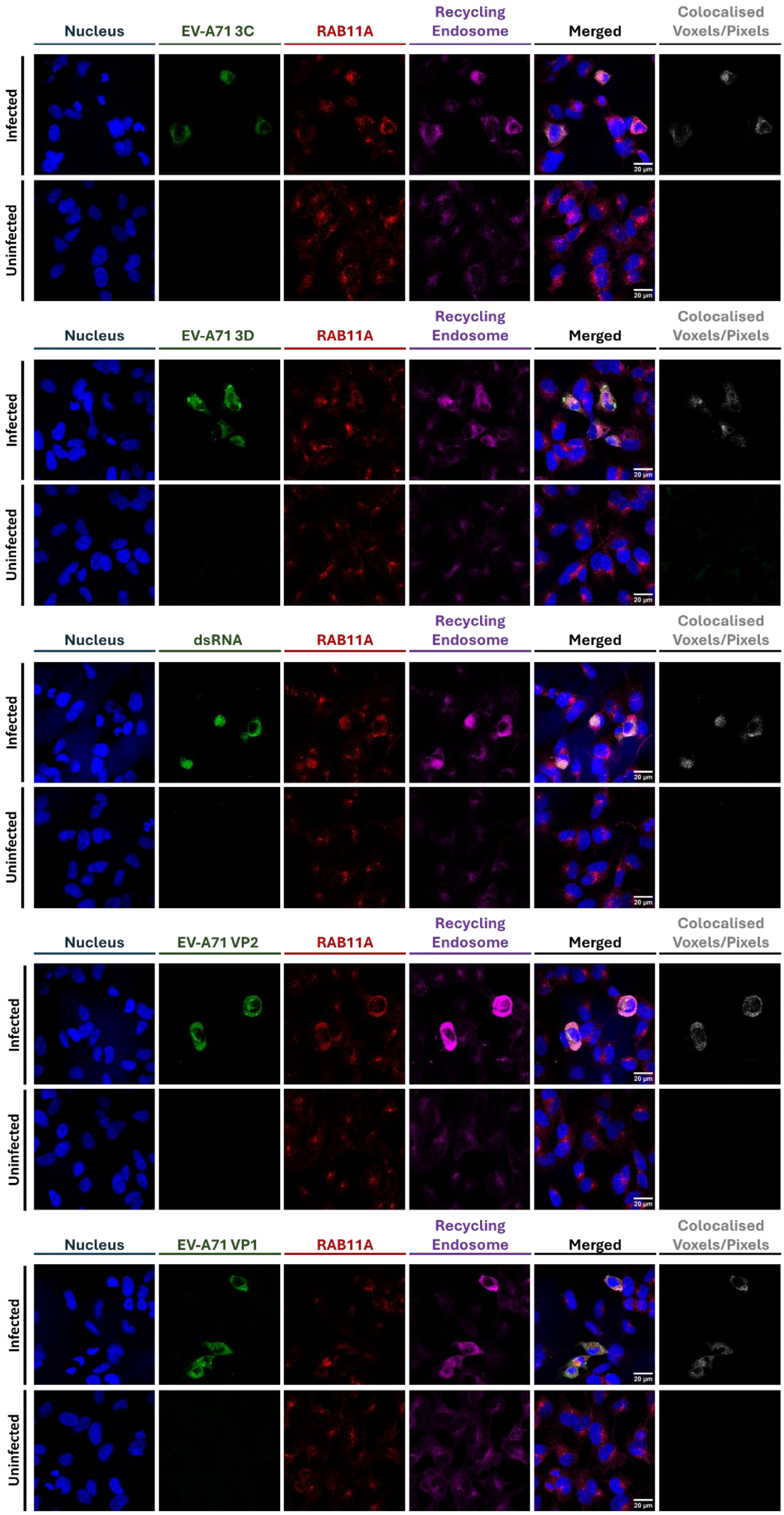
Nucleus
EV-A71 3C
RAB11A
Recycling Endosome
Merged
Colocalised Voxels/Pixels
Infected
Uninfected
Nucleus
EV-A71 3D
RAB11A
Recycling Endosome
Merged
Colocalised Voxels/Pixels
Infected
Uninfected
Nucleus
dsRNA
RAB11A
Recycling Endosome
Merged
Colocalised Voxels/Pixels
Infected
Uninfected
Nucleus
EV-A71 VP2
RAB11A
Recycling Endosome
Merged
Colocalised Voxels/Pixels
Infected
Uninfected
Nucleus
EV-A71 VP1
RAB11A
Recycling Endosome
Merged
Colocalised Voxels/Pixels
Infected
Uninfected
20 µm

D.

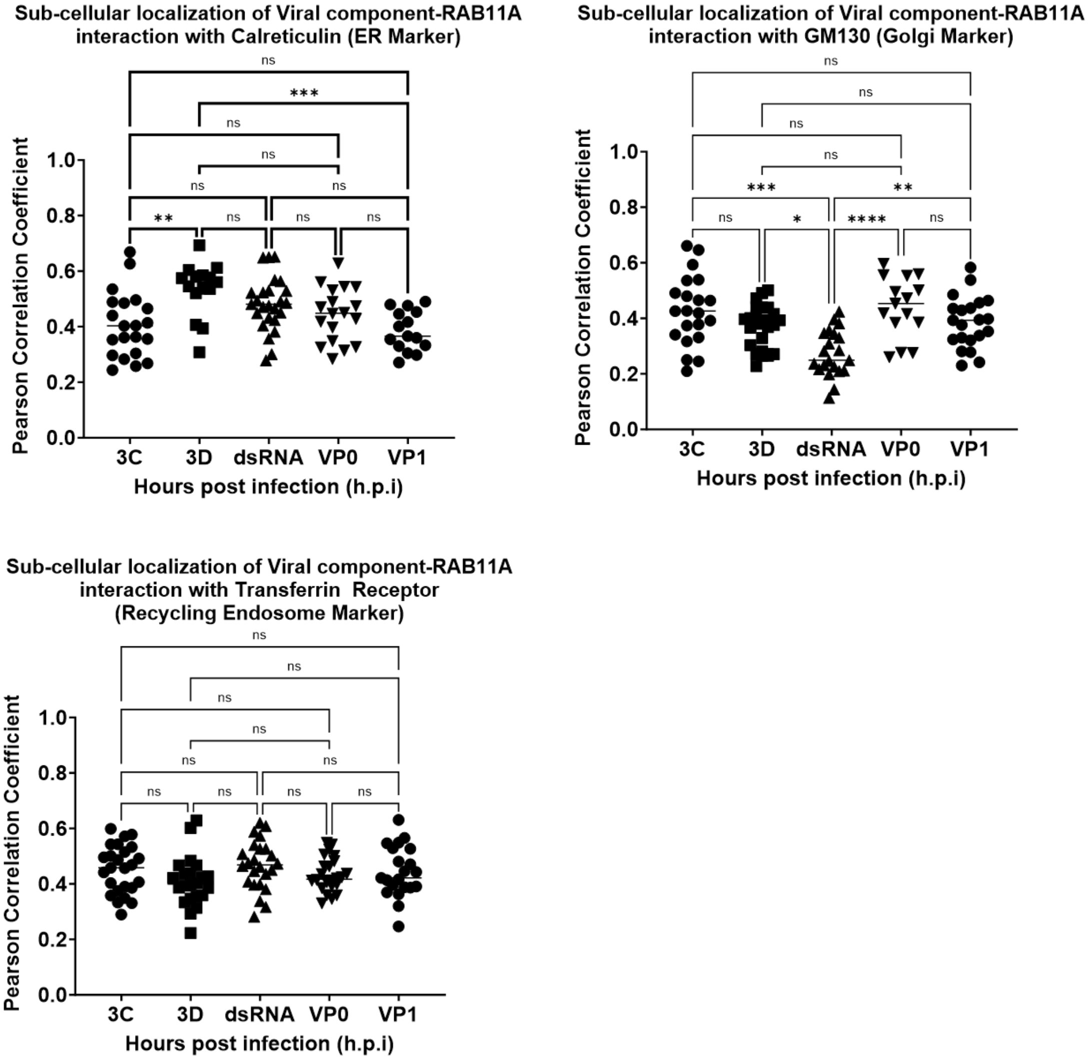

Figure S7. Interactions between RAB11A and viral proteins VP1, VP2, 3D, 3C, or dsRNA in various subcellular compartments at 6 hours post-infection.

SH-SY5Y cells were infected with EV-A71 S41 (MOI 0.1). At the indicated time points post-infection, the cells were fixed and permeabilized, followed by staining with anti-RAB11A and anti-VP2, VP1, 3C, 3D or dsRNA primary antibodies, and labelled secondary antibodies. Cells were then further stained with antibodies specific to compartment markers calreticulin for endoplasmic reticulum **(A)**, GM130 for Golgi apparatus **(B)** and transferrin receptor (Tfr) for small recycling endosomes **(C)** prior to DAPI staining. Confocal images were captured under 100X objective and were analyzed using Fiji software to determine the voxels/pixels that represent three channels (green, red, magenta) co-localization. Briefly, a mask representing the co-localization of RAB11A and each viral component was delineated and subsequently overlayed with compartment marker signals to generate the 'co-localized voxels' images shown on the far right column. **(D)** Pearson correlation coefficient (PCC) values were computed using Fiji software. PCC value of 0 = no co-localization; 0.1 – 0.3 = weak co-localization; 0.3 – 0.5 = moderate co-localization; 0.5-1 = strong co-localization. Statistical analysis was performed using Kruskal-Wallis test with Dunn correction against siNTC treatment (*p<0.05, **p<0.01, ***p<0.001, ****p<0.0001).

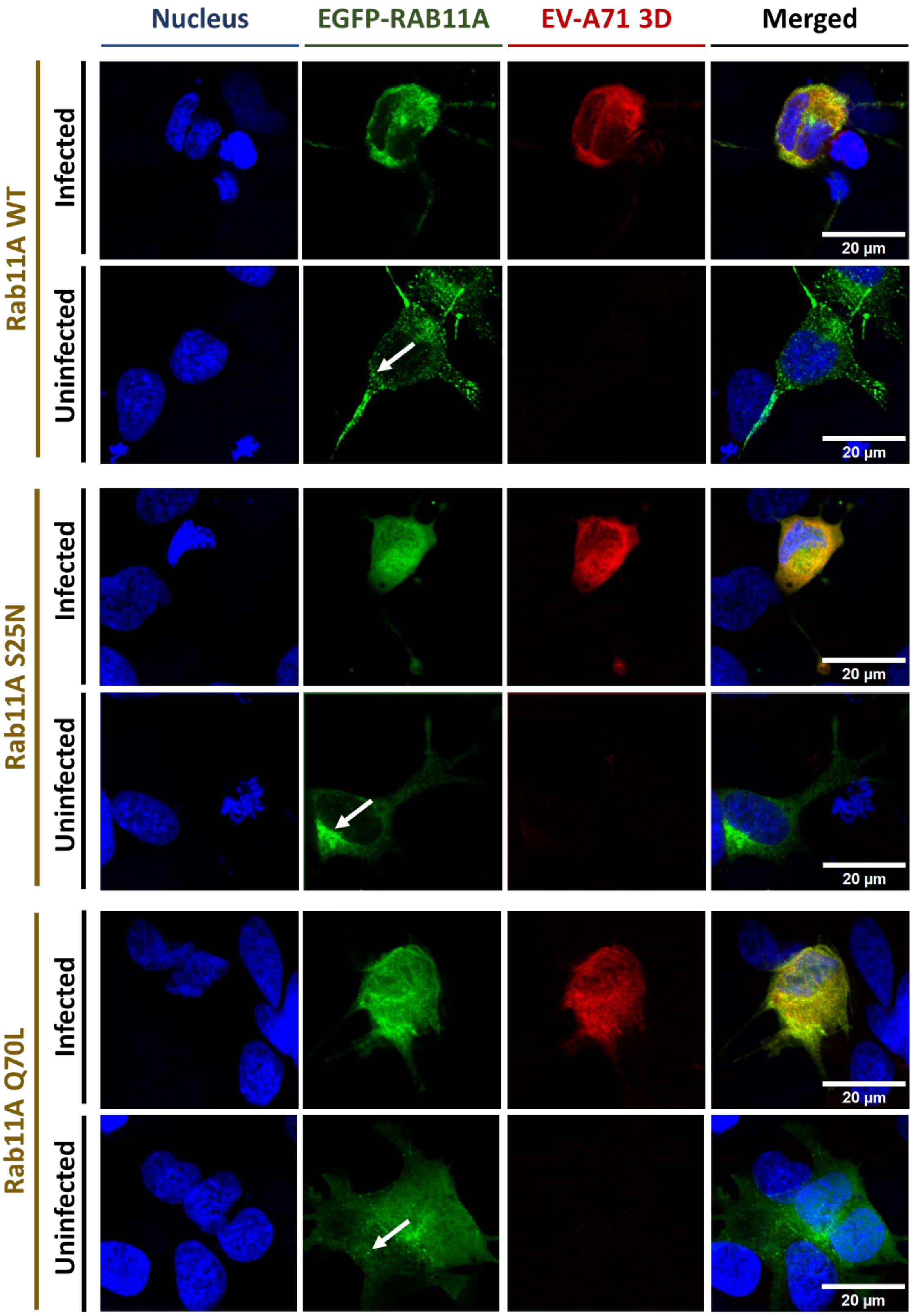

Figure S8. Distribution patterns of overexpressed RAB11A WT, DN (RAB11A S25N) and CA (RAB11A Q70L) in infected and uninfected SH-SY5Y cells. Images taken from Fig. S8 panel B were further zoomed in (200%) using Fiji software. White arrows indicate specific distribution patterns that were lost in infected cells.

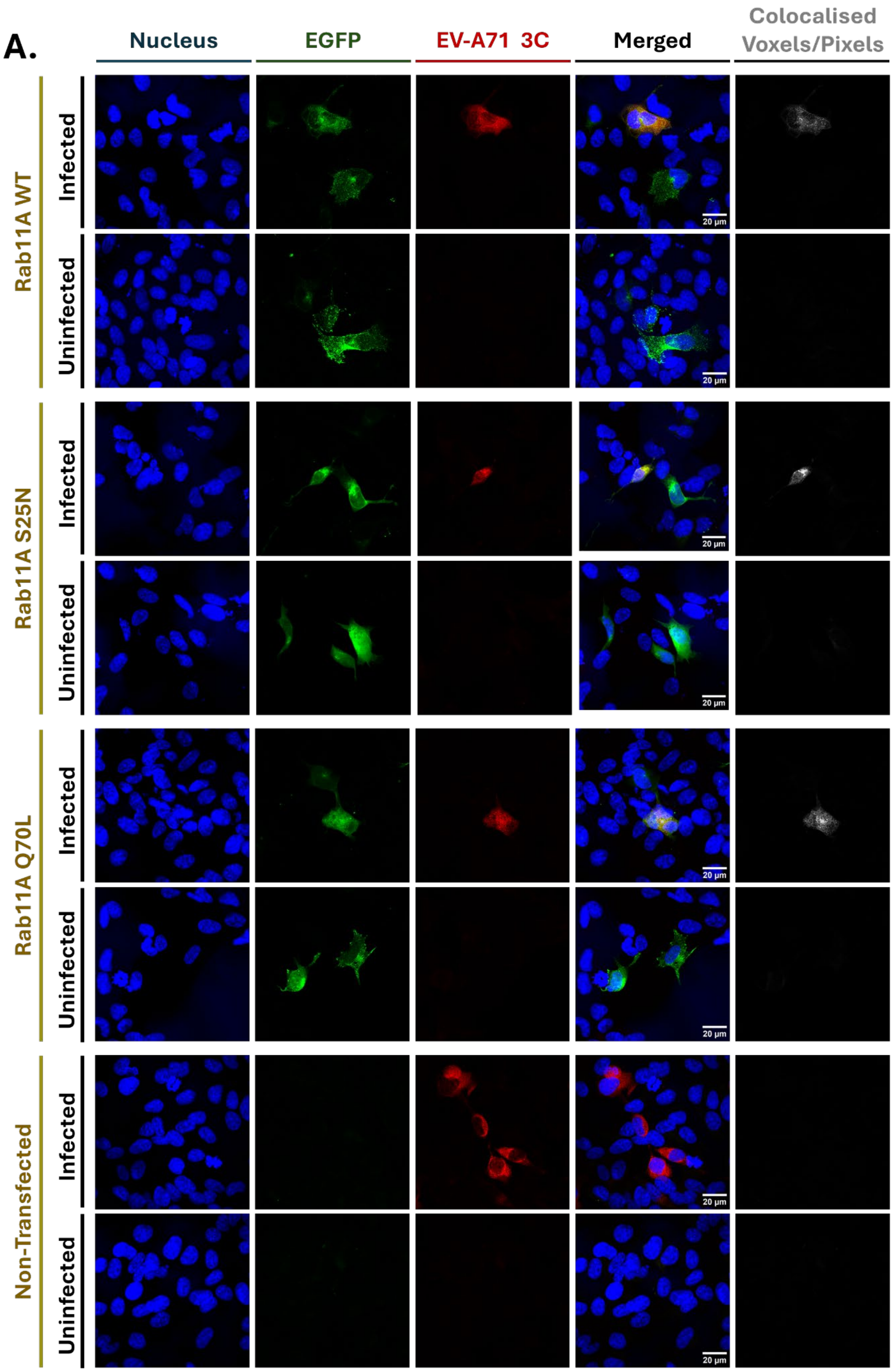
A.
Nucleus
EGFP
EV-A71 3C
Merged
Colocalised Voxels/Pixels
Rab11A WT
Rab11A S25N
Rab11A Q70L
Non-Transfected
Infected
Uninfected
20 µm

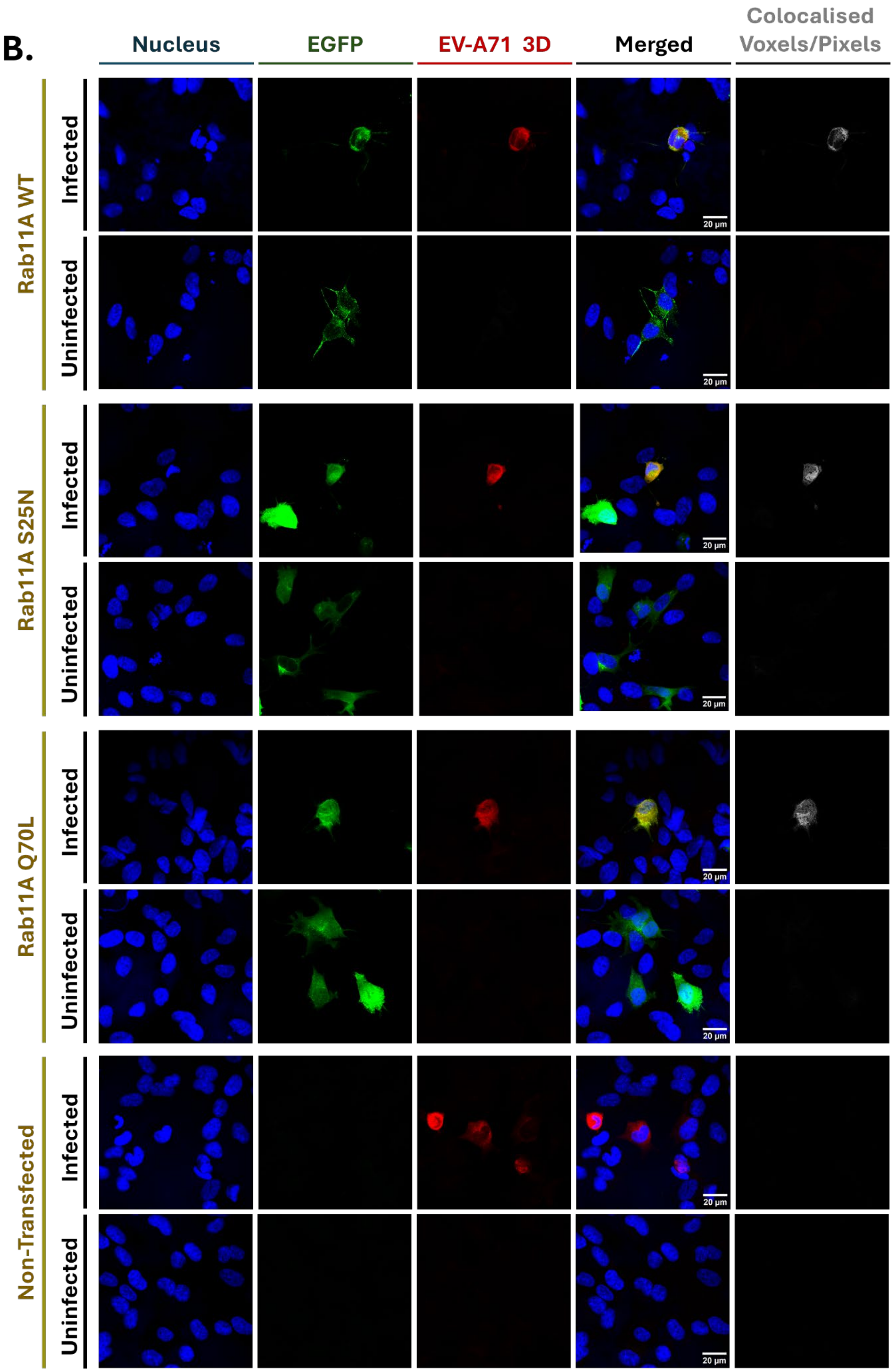
B.
Nucleus
EGFP
EV-A71 3D
Merged
Colocalised Voxels/Pixels
Rab11A WT
Rab11A S25N
Rab11A Q70L
Non-Transfected
Infected
Uninfected
20 µm

C.

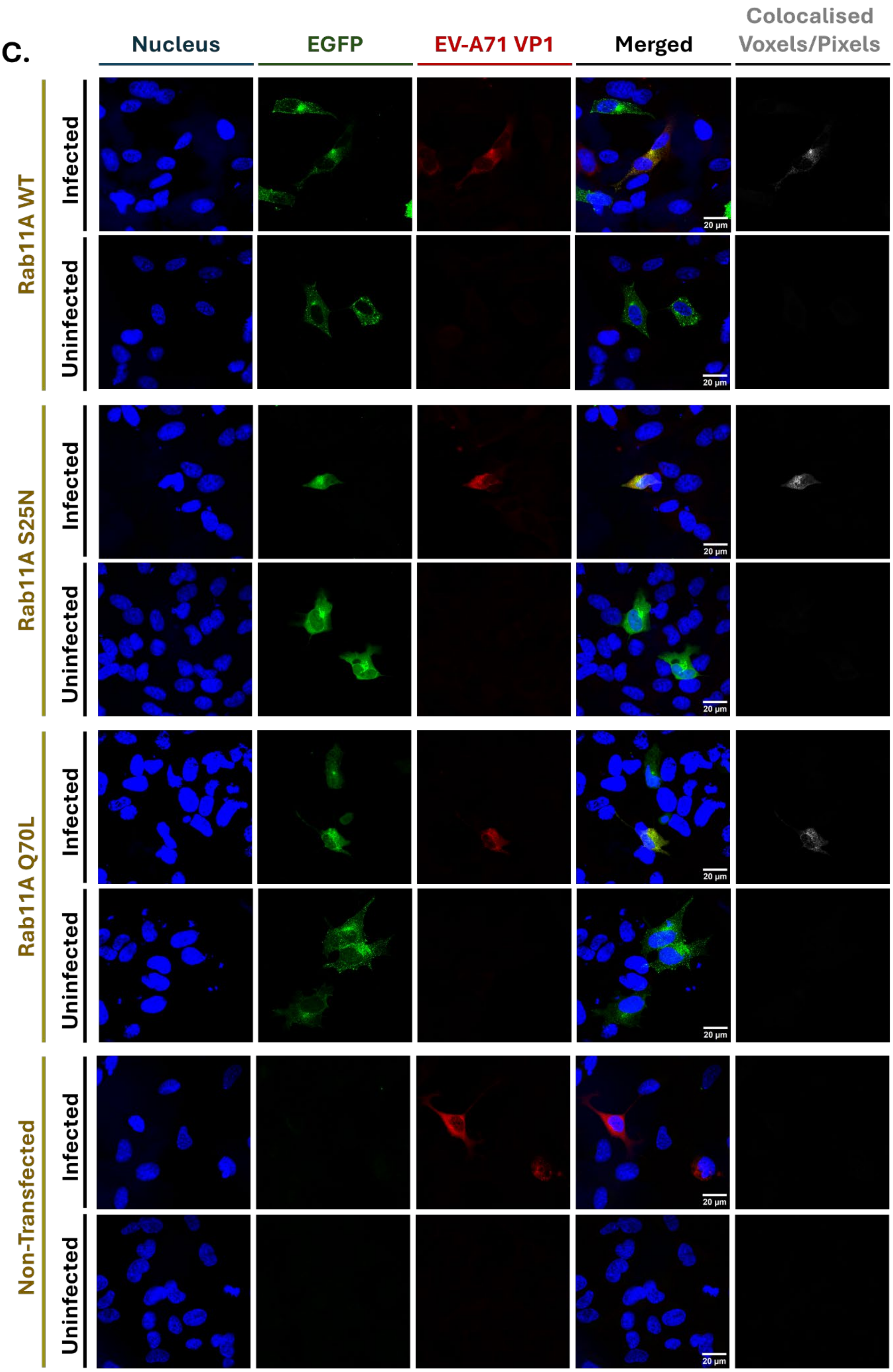

Nucleus
EGFP
EV-A71 VP1
Merged
Colocalised Voxels/Pixels
Rab11A WT
Rab11A S25N
Rab11A Q70L
Non-Transfected
Infected
Uninfected
Infected
Uninfected
Infected
Uninfected
Infected
Uninfected
20 µm

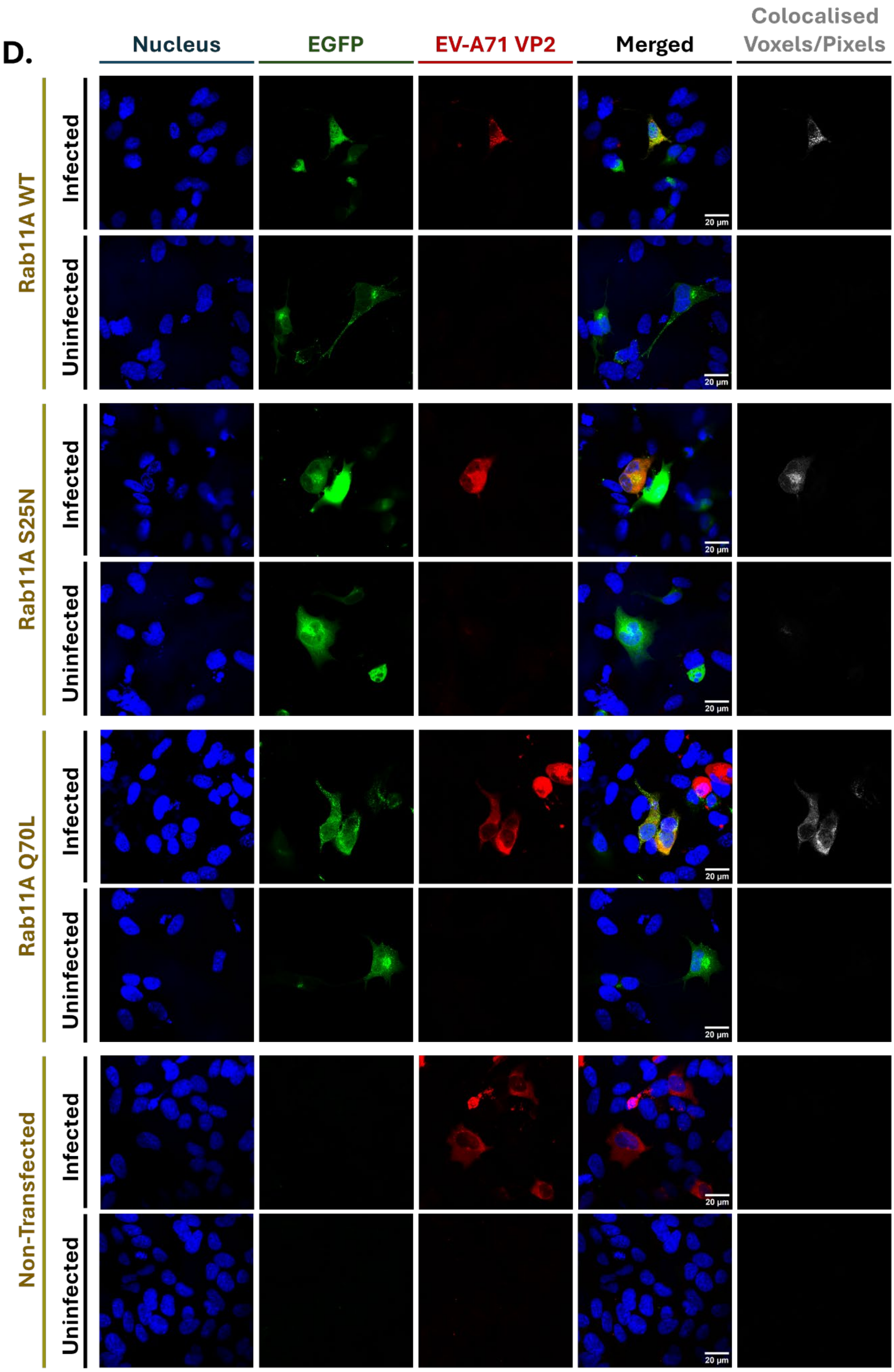

D.
Nucleus
EGFP
EV-A71 VP2
Merged
Colocalised Voxels/Pixels
Rab11A WT
Rab11A S25N
Rab11A Q70L
Non-Transfected
Infected
Uninfected
20 μm

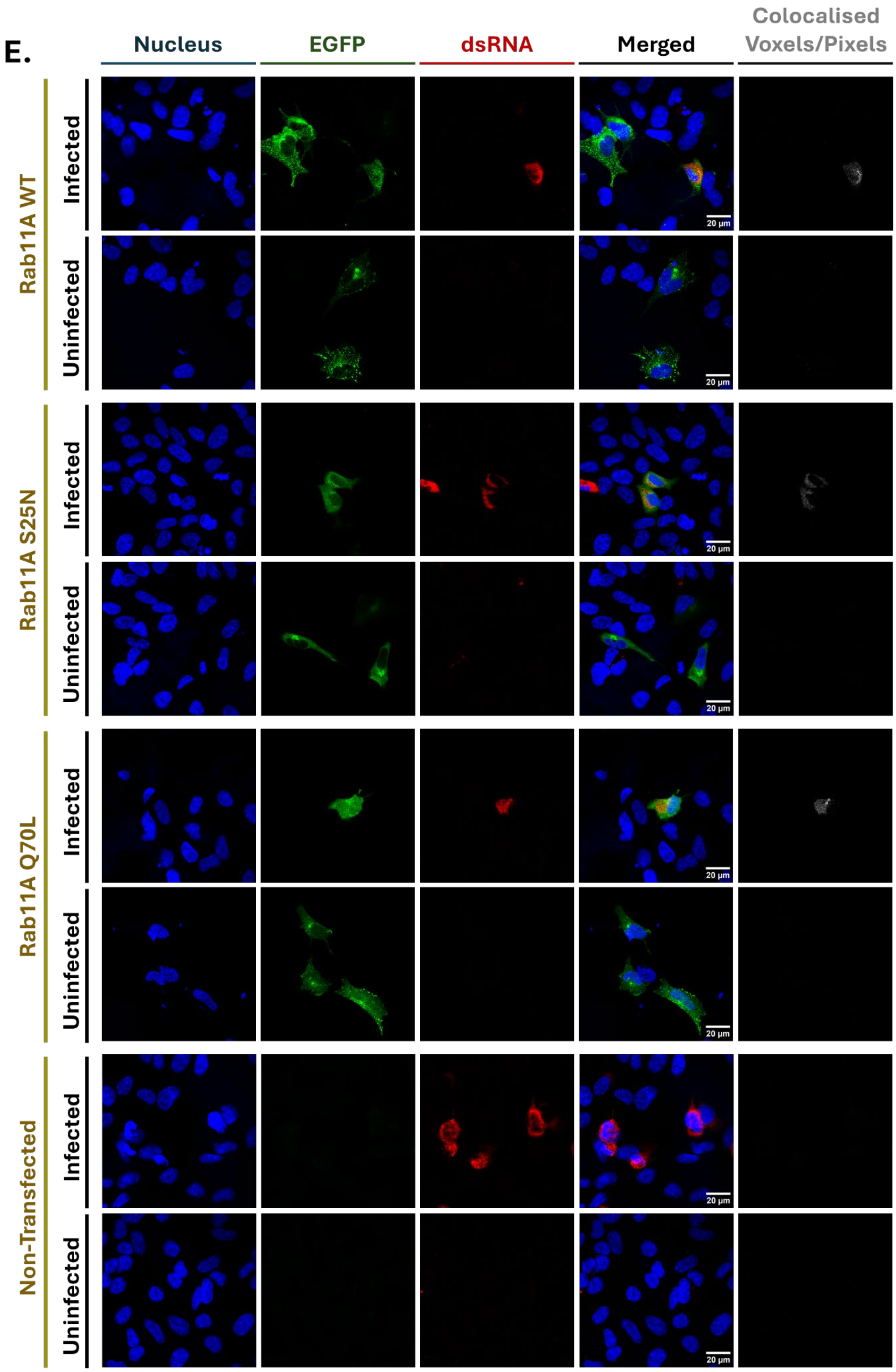
E.
Nucleus
EGFP
dsRNA
Merged
Colocalised Voxels/Pixels
Rab11A WT
Infected
Uninfected
Rab11A S25N
Infected
Uninfected
Rab11A Q70L
Infected
Uninfected
Non-Transfected
Infected
Uninfected
20 µm

F.

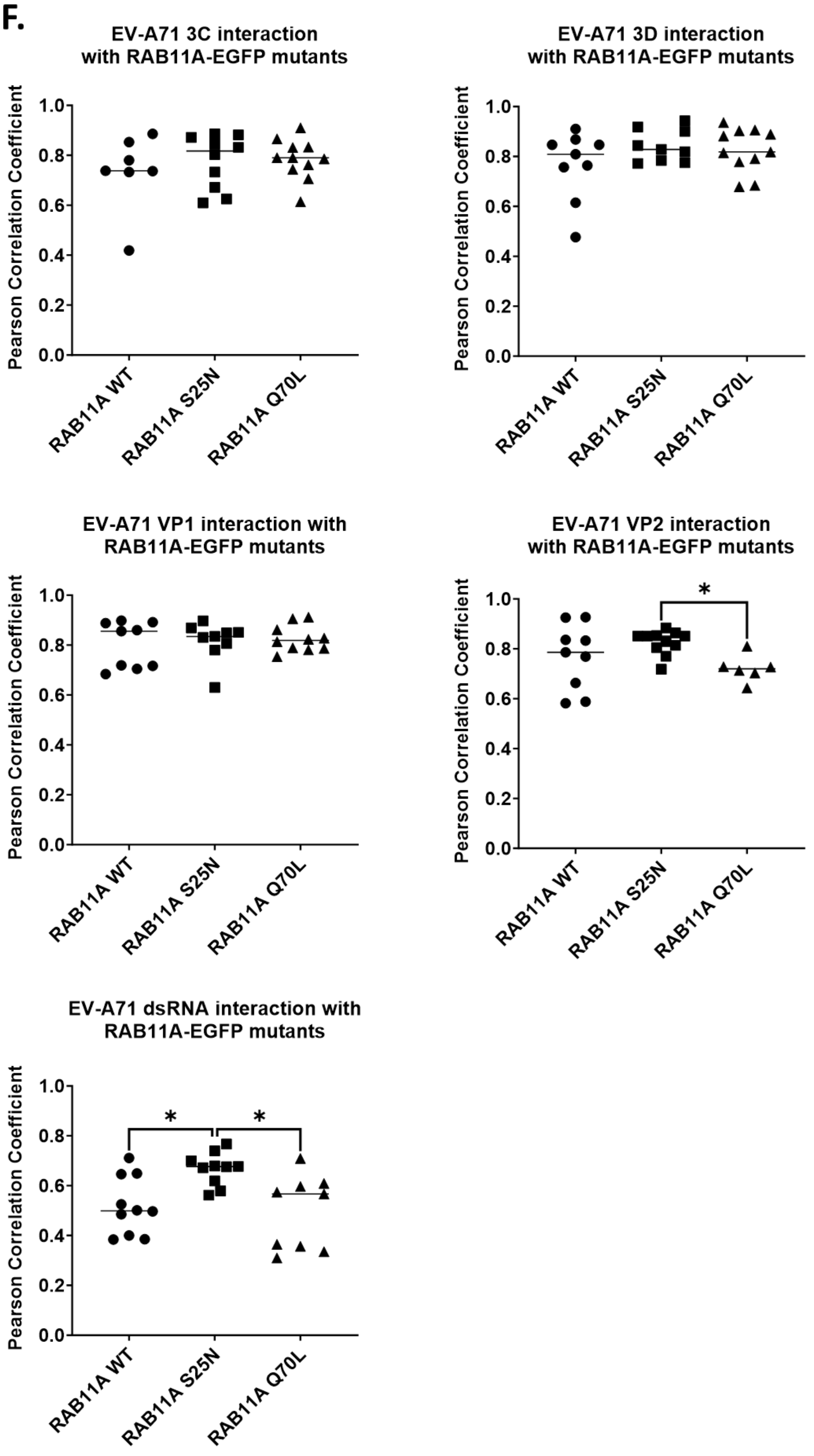

Figure S9. Co-localization between RAB11AWT, RAB11AS25N and RAB11AQ70L with viral components.
SH-SY5Y cells over-expressing EGFP-RAB11AWT, EGFP-RAB11AS25N or EGFP-RAB11AQ70L, were infected with EV-A71 S41 (MOI 0.1). At 12 h.p.i , these cells were fixed and permeabilized, before staining with **(A)** anti-3C, **(B)** anti-3D, **(C)** anti- VP2, **(D)** anti-VP1 or **(E)** anti-dsRNA. Nuclei were stained with DAPI. Confocal images were captured under 100X objective and analyzed using Fiji software to determine the co-localized voxels that represent co-localization of red and green channels. **(F)** Pearson correlation coefficient (PCC) values were computed using Fiji software. PCC value of 0 = no co-localization; 0.1 – 0.3 = weak co-localization; 0.3 – 0.5 = moderate co-localization; 0.5-1 = strong co-localization. Statistical analysis was performed using Kruskal-Wallis test wit Dunn correction against siNTC treatment (*p<0.05, **p<0.01, ***p<0.001, ****p<0.0001).

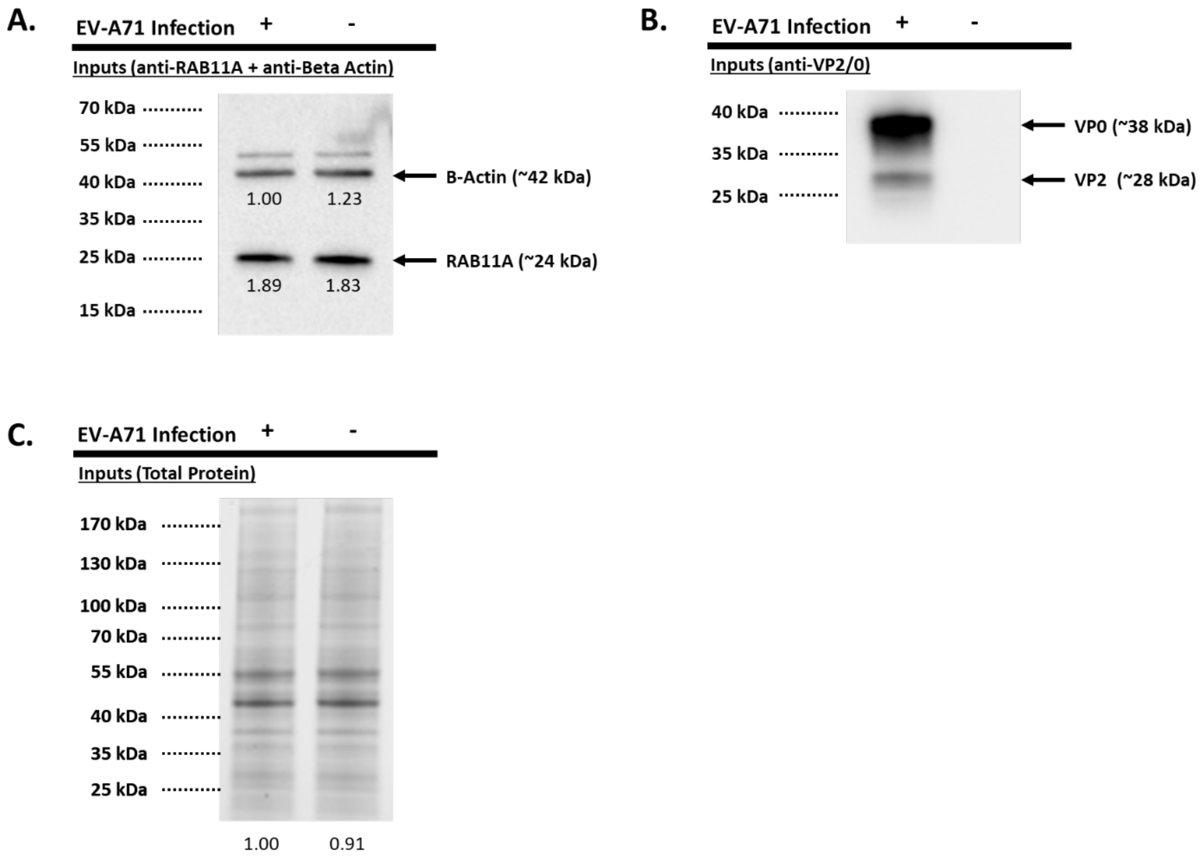

Figure S10. Total protein content and RAB11A expression in input samples prior to Co-IP for mass spectrometry (Fig. 9). 10ug of each input sample was used for gel electrophoresis. **(A&B)** Western blot analysis using anti-RAB11A, anti-VP2 and anti- B-actin. **(C)** TGX stain-free gel to visualize the total protein content in each sample.

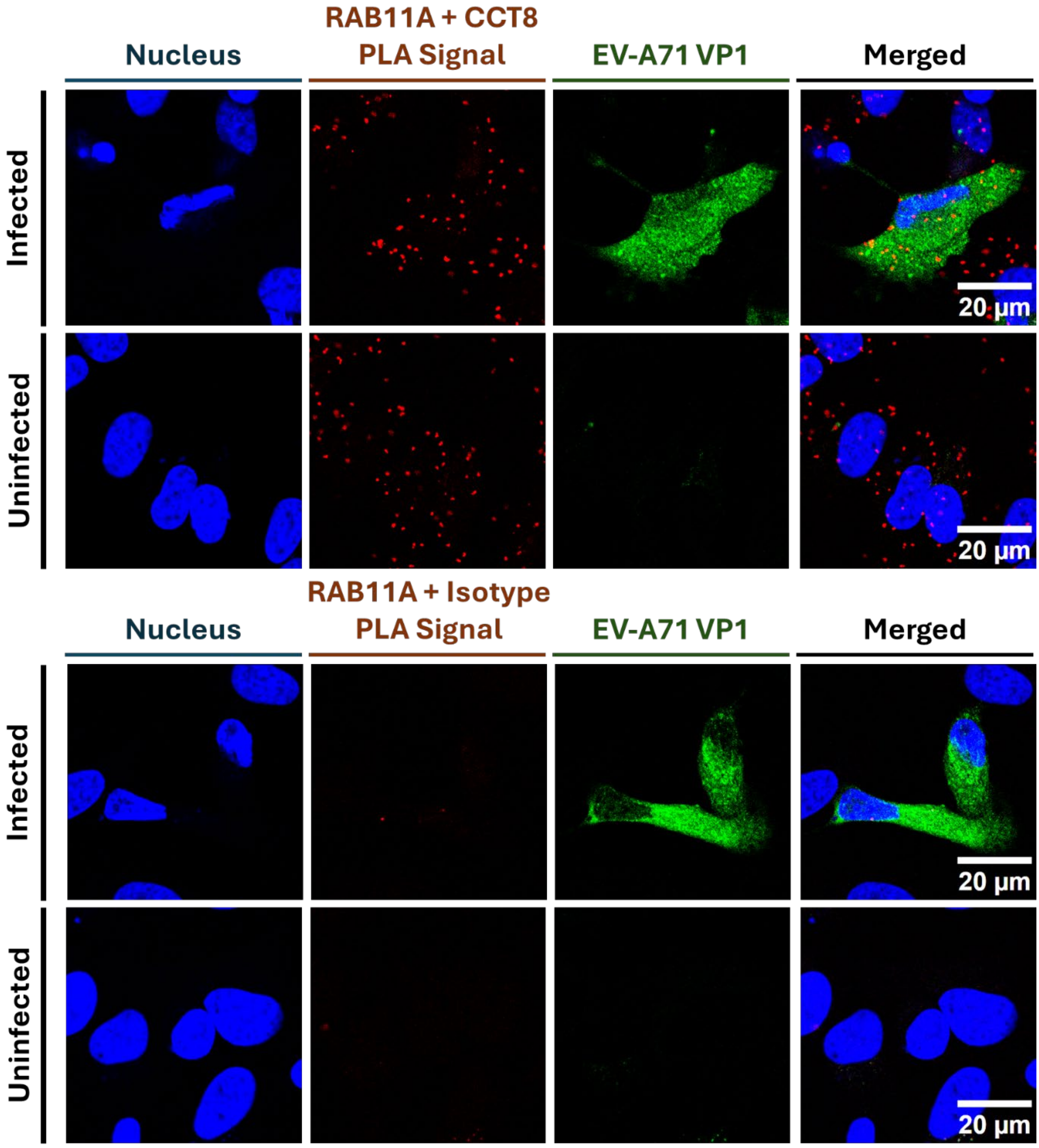

Figure S11. RAB11A and CCT8 co-localization and interaction.

Co-localization and interaction between RAB11A with CCT8 was assessed by proximity ligation assay (PLA). **(A)** SH-SY5Y cells were infected with EV-A71 S41 (MOI 0.1). At 24 h.p.i., the cells were fixed and permeabilized before staining with anti-RAB11A paired with either anti-CCT8 or IgG isotype control antibodies. The cells were then stained with secondary antibodies conjugated with DNA probes. Ligation and polymerase chain reaction were then carried out for signal amplification. These cells were further stained using in-house conjugated EV-A71 VP1 antibody to identify infected cells. Nuclei were stained with DAPI. Images were captured at 100X magnification under Olympus FV3000 confocal microscope. Images were further zoomed in by 150% using Fiji software.
